# Panthenol Citrate Biomaterials Accelerate Wound Healing and Restore Tissue Integrity

**DOI:** 10.1002/adhm.202301683

**Published:** 2023-06-25

**Authors:** Huifeng Wang, Chongwen Duan, Rebecca L. Keate, Guillermo A. Ameer

**Affiliations:** ^1^ Department of Biomedical Engineering Northwestern University Evanston IL 60208 USA; ^2^ Center for Advanced Regenerative Engineering Northwestern University Evanston IL 60208 USA; ^3^ Department of Surgery Feinberg School of Medicine Northwestern University Chicago IL 60611 USA; ^4^ Chemistry of Life Processes Institute Northwestern University Evanston IL 60208 USA; ^5^ Simpson Querrey Institute Northwestern University Chicago IL 60611 USA; ^6^ International Institute for Nanotechnology Northwestern University Evanston IL 60208 USA

**Keywords:** diabetic wound healing, panthenol citrate, provitamin B_5_, regenerative engineering and medicine

## Abstract

Impaired wound healing is a common complication for diabetic patients and effective diabetic wound management remains a clinical challenge. Furthermore, a significant problem that contributes to patient morbidity is the suboptimal quality of healed skin, which often leads to reoccurring chronic skin wounds. Herein, a novel compound and biomaterial building block, panthenol citrate (PC), is developed. It has interesting fluorescence and absorbance properties, and it is shown that PC can be used in soluble form as a wash solution and as a hydrogel dressing to address impaired wound healing in diabetes. PC exhibits antioxidant, antibacterial, anti‐inflammatory, and pro‐angiogenic properties, and promotes keratinocyte and dermal fibroblast migration and proliferation. When applied in a splinted excisional wound diabetic rodent model, PC improves re‐epithelialization, granulation tissue formation, and neovascularization. It also reduces inflammation and oxidative stress in the wound environment. Most importantly, it improves the regenerated tissue quality with enhanced mechanical strength and electrical properties. Therefore, PC could potentially improve wound care management for diabetic patients and play a beneficial role in other tissue regeneration applications.

## Graphical abstract

A compound and biomaterial building block, panthenol citrate (PC), is reported. PC can be used in soluble form as a cleansing solution and as a hydrogel dressing to address diabetes‐impaired wound healing. PC exhibits antioxidant, antibacterial, anti‐inflammatory, and pro‐angiogenic properties, and promotes keratinocyte and dermal fibroblast migration and proliferation. PC improves regenerated tissue quality, enhancing mechanical and electrical properties.

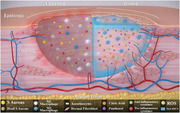

## 1 Introduction

Diabetes is a growing epidemic in the western world and diabetic foot ulcers (DFU) are the most common diabetes‐related complication leading to hospitalization and lower limb amputation.^[^ ^1^, ^2^ ^]^ Approximately 34% of diabetics will develop a DFU in their lifetime, with 15–20% of those becoming chronic or non‐healing wounds.^[^ ^3^, ^4^ ^]^ In contrast to normal wounds, healing in diabetic wounds is impaired due to persistent oxidative stress, abnormal inflammatory response dynamics, impaired vascularization, and innervation, among other problems that may be patient‐specific.^[^ ^5^, ^6^, ^7^, ^8^, ^9^, ^10^ ^]^ In addition to delayed wound healing, suboptimal physical properties, such as mechanical strength and electrophysiological properties of regenerated tissue often lead to recurrence or reopening of the wound. It has been reported that 40% of patients with DFUs experience a recurrence of the wound within 1 year after the ulcer heals and that number increases to 60% and 65% within 3 and 5 years, respectively.^[^ ^3^ ^]^ Of note, 71–85% of recurring DFUs can result in amputation.^[^ ^11^ ^]^ Therefore, there is a high demand for wound treatments that not only accelerate wound closure, but also enhance the quality of regenerated tissue to improve the outcomes of wound care management for diabetic patients.

Various approaches have been developed to treat impaired diabetic wounds, such as the administration of cytokines and growth factors,^[^ ^12^, ^13^, ^14^ ^]^ cell‐based therapies,^[^ ^15^, ^16^ ^]^ or negative pressure wound therapy (NPWT).^[^ ^17^ ^]^ In recent years, many studies have focused on the development of biomimetic scaffold systems that are associated with peptide‐, cytokine‐, or growth factor‐based therapeutics.^[^ ^18^, ^19^, ^20^ ^]^ However, conflicting evidence was found in these studies leading to only a few randomized controlled trials.^[^ ^21^, ^22^ ^]^ Moreover, growth factor‐based therapies do not prevent recurrence of DFUs.^[^ ^23^ ^]^ Cell‐based therapy is considered advantageous over growth factors for DFU treatment because cells can regulate tissue regeneration in a comprehensive manner by refining the microenvironment of the wound site.^[^ ^24^ ^]^ A study conducted on patients with DFUs using mesenchymal stromal cells (MSCs) revealed good treatment tolerance and a shortened healing time. Furthermore, patients did not experience recurring DFUs followed by amputation for three years after the treatment.^[^ ^25^ ^]^ Although stem cell applications to DFUs have entered the clinical stage, concerns regarding safety and scalability remain.^[^ ^26^, ^27^ ^]^ Biomaterials have gained significant attention for their capacity to function as a physical barrier and provide a microenvironment that promotes wound healing.^[^ ^28^, ^29^, ^30^ ^]^ Despite the wide variety of biomaterials that have been studied in several preclinical models to address treatment of DFUs, study outcomes focus on the histopathology analyses of the healed tissue and do not address or investigate parameters associated with the function or durability of the regenerated tissue such as mechanical and electrophysiological properties. Therefore, there is a need to also understand the degree to which the protective function of the tissue is restored, which may provide insight on the potential durability of the treatment.

Herein, we report the synthesis and characterization of panthenol citrate (PC) and its versatile use in soluble form as a wash or cleansing solution and as a regenerative dressing to address impaired wound healing in diabetes. PC is synthesized by reacting citric acid with panthenol and it has interesting optical, chemical, and biological properties that are conducive to protecting and regenerating skin tissue. Citric acid, which is naturally produced in the body, is widely available and has an established safety record in both consumer and professional products.^[^ ^31^, ^32^, ^33^, ^34^, ^35^ ^]^ Citric acid has been utilized to synthesize biomaterials by reacting it with diols and a version of these biomaterials is now used in implantable medical devices.^[^ ^36^ ^]^ Panthenol is a natural amine‐containing diol that is rapidly oxidized by the body to pantothenic acid, also known as vitamin B_5_. Its use in the cosmetics industry is supported by research indicating that D‐Panthenol possesses moisturizing and anti‐inflammatory properties when applied to the skin.^[^ ^37^, ^38^, ^39^ ^]^ We show that PC exhibits antioxidant, antibacterial, anti‐inflammatory, and proangiogenic properties that can accelerate wound closure in diabetes and restore the mechanical and electrophysiological properties of skin. When used in solution and hydrogel forms, PC promotes keratinocyte and dermal fibroblast migration and proliferation, resulting in improved re‐epithelialization, granulation tissue formation, and accelerated wound closure. Most importantly, the mechanical strength and electrophysiological properties of the regenerated skin are significantly enhanced. To the best of our knowledge, this is the first study to demonstrate that the histological and physical properties of regenerated skin, following treatment with both monomeric and polymeric forms of a compound, are significantly improved. Our approach involves using PC alone as a wound cleansing solution or formulating it into a thermoresponsive biomacromolecule for use as a regenerative dressing. Therefore, PC is a building block for advanced therapies for tissue regeneration that is expected to improve wound care management for diabetic patients.

## 2 Results and Discussion

### 2.1 Panthenol Citrate Is a Photoluminescent Compound that Can Be Dissolved in an Aqueous Solution or Incorporated into a Thermoresponsive Hydrogel

**Figure 1:**
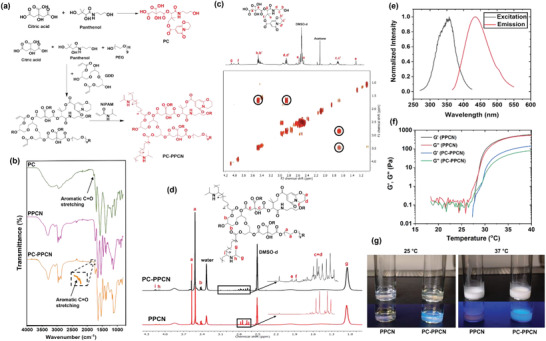
Synthesis and characterization of PC and PC‐PPCN. a) Scheme of the chemical reactions to synthesize PC and PC‐PPCN. b) FT‐IR spectra of PC, PPCN, and PC‐PPCN. c) ^1^H NMR and 2D NMR spectra of PC. d) ^1^H NMR spectra of PPCN and PC‐PPCN. e) Photoluminescent properties of PC. f) The thermally‐induced phase change of PPCN and PC‐PPCN. The crossover temperature of storage modulus (*G*′) and loss modulus (*G*″) defined their LCST as 28 °C for PPCN and 30 °C for PC‐PPCN. g) Digital image of PPCN and PC‐PPCN at 25 °C and 37 °C, respectively. PPCN and PC‐PPCN are liquids at 25 °C and become gels at 37 °C. PC‐PPCN emits blue fluorescence under UV exposure at 365 nm.

The panthenol citrate (PC) compound was synthesized via a thermal condensation reaction between panthenol and citric acid. PC was also formulated into a thermoresponsive hydrogel form poly (panthenol citrate polyethylene glycol citrate co‐N‐isopropylacrylamide) (PC‐PPCN) by reacting citric acid, panthenol, polyethylene glycol (PEG), and glycerol 1,3‐diglycerolate diacrylate (GDD) via thermal condensation and subsequent free radical polymerization with NIPAAM (**Figure** **1**a). PPCN without the panthenol was synthesized as previously described to serve as a reference material for evaluating the effects of incorporating PC into a biomacromolecule.^[^ ^40^ ^]^ The chemical structures of PC and PC‐PPCN were confirmed by both ^1^H NMR and FT‐IR spectroscopy (Figure 1b–d). The molar ratios of panthenol, citric acid, PEG, GDD, and NIPAAm in PC‐PPCN were determined to be 1.2:1.6:2.1:1:14.3 from ^1^H NMR. The amount of PC in PC‐PPCN was 10.6 mol%. The peaks at 1.68, 2.15, 2.85, and 3.48 ppm in ^1^H NMR and the black circles in the 2D NMR spectrum were attributed to the proton on the aromatic structure of PC. This was also confirmed by the appearance of a peak at 1780 cm^−1^ in the FT‐IR spectrum corresponding to aromatic C=O stretching. Due to the conjugated ring structure, PC absorbed UV at 350 nm and emitted blue fluorescence at 450 nm, which can be potentially used in bioimaging applications^[^ ^41^ ^]^ (Figure 1e). This fluorescence is consistent with the findings of others who proposed the possible mechanism for the formation of the fluorophore.^[^ ^41^, ^42^ ^]^ We found that the molecular weight of PC is 462 g mol^−1^ as per mass spectroscopy and the number average molecular weight of PPCN is 16 314 g mol^−1^ as per gel permeation chromatography (GPC) (Figure S1, Supporting Information). We could not determine the molecular weight of PC‐PPCN using mass spectroscopy or GPC likely due to the strong *π*–*π* stacking and hydrogen bonding intramolecular/intermolecular interactions between the polymer chains. Nevertheless, we determined that nanoparticles of PPCN and PC‐PPCN in water had similar hydrodynamic diameters (166.8 and 148.7 nm, respectively) suggesting that they have similar molecular sizes (Figure S1, Supporting Information). The thermal transition behavior and thermal stability of PPCN and PC‐PPCN were evaluated by DSC and TGA (Figure S2, Supporting Information). Both PPCN and PC‐PPCN were completely amorphous polymers with a glass transition temperature (Tg) at 91.8 and 75.1 °C and highly thermostable with a decomposition temperature of ≈200 °C due to their crosslinked structures. The lower critical solution temperature for PPCN and PC‐PPCN was 28 and 30 °C, respectively, allowing application to the wound as a liquid that transitions to a conformal gel at body temperature to form a stable dressing (Figure 1f; Video S1, Supporting Information). This phase change also enables easy dressing removal from the wound without causing any secondary injury by applying cold saline solution. The introduction of panthenol slightly increased the LCST and lowered the storage modulus owing to the hydrophilicity of panthenol. Thermoresponsive hydrogels offer several advantages for wound healing. Their ability to respond to temperature changes makes them adaptable to the dynamic environment of the wound bed.^[^ ^43^ ^]^ The transition from a liquid to a solid gel state upon exposure to body temperature allows these hydrogels to conform to the wound bed.^[^ ^44^ ^]^

### 2.2 PC and PC‐PPCN Exhibit Antioxidant and Antibacterial Properties

**Figure 2:**
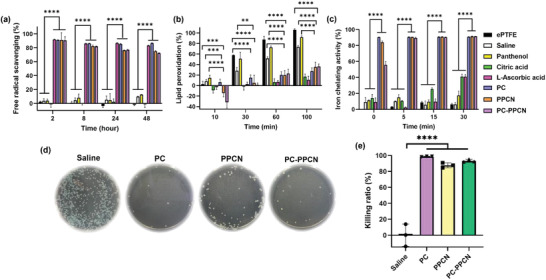
Antioxidant and antibacterial properties of PC and PC‐PPCN. a–c) Antioxidant properties of PC, PC‐PPCN, and PPCN were confirmed via ABTS free radical scavenging, *β*‐carotene lipid peroxidation, and Fe^2+^ ion chelating assays. All three materials are capable of scavenging free radicals, inhibiting lipid peroxidation, and chelating Fe^2+^ ions. d) Digital images of *S. aureus* colonies on culture plates after incubation with saline, PC, PPCN, and PC‐PPCN. e) Quantification of the images in (d) describing the resulting bacteria killing ratio against *S. aureus*. All data are presented as mean ± SD (*n* = 3 for antioxidant assays; ns: not significant; *p < 0.05; **p < 0.01; ***p < 0.001, ****p < 0.0001).

Citrate‐based biomaterials (CBBs) exhibit antioxidant properties.^[^ ^40^ ^]^ Their capacity to scavenge free radicals, prevent lipid peroxidation, and chelate iron ions likely play a significant role in their biocompatibility and reported low tissue inflammatory response.^[^ ^45^ ^]^ Therefore, the antioxidant properties of PC and PC‐PPCN were evaluated by ABTS free radicals scavenging, *β*‐carotene bleaching, and ferrous iron chelating assay. PC, PPCN, and PC‐PPCN rapidly stabilized free radicals, which exhibited above 80% free radical scavenging activity within 48 h, similar to the well‐known antioxidant l‐ascorbic acid. ePTFE, saline, panthenol, and citric acid did not demonstrate ABTS free radical scavenging ability (**Figure** **2**a). PC, PC‐PPCN, as well as PPCN, citric acid, and l‐ascorbic acid inhibited *β*‐carotene lipid peroxidation by scavenging superoxide free radicals, with a fivefold reduction of lipid peroxidation when compared to ePTFE, saline and panthenol (Figure 2b). Many radical reactions that occur in the body are initiated by ferrous ions (Fe^2+^).^[^ ^46^ ^]^ Due to the number of carboxylate groups, CBBs can chelate multivalent transition metal ions. PC, PPCN, and PC‐PPCN chelated 90% ferrous ions within 5 min, which was 5 times more than the negative controls and twice that of citric acid and l‐ascorbic acid (Figure 2c). These findings show that panthenol or citric acid, on their own, are not efficient radical scavengers. However, both PC and PC‐PPCN rapidly stabilize free radicals, likely due to the formation of aromatic rings.

Bacterial infection in the diabetic wound is another major reason for delayed wound healing.^[^ ^47^ ^]^ Therefore, wound dressings with antibacterial capability will be beneficial for clinical applications. Citric acid has been used as an antibacterial preservative due to its ability to inhibit bacterial growth by chelating calcium and magnesium ions and disrupting cell membranes.^[^ ^48^, ^49^ ^]^ Panthenol and its derivatives have also shown their capacity to inhibit the growth of *Staphylococcus aureus*.^[^ ^50^ ^]^ The antibacterial properties of PC and PC‐PPCN, as well as PPCN, panthenol, citric acid, and calcium pantothenate (vitamin B_5_) were investigated using *S. aureus*, the most common bacteria found in wounds resulting in the most complications.^[^ ^51^ ^]^ Bacteria suspensions were spread on agar plates and incubated overnight. Colony‐forming units (CFU) were photographed to evaluate the antibacterial activity of PC and PC‐PPCN.  Relative to saline, PC, PPCN, and PC‐PPCN exhibited 98.6%, 87.7%, and 93.3% reduction of *S. aureus* colonies, respectively (Figure 2d,e). The killing ratios of citric acid, panthenol, and calcium pantothenate against *S. aureus* were 99.6%, 95.9%, and 95.0%, respectively (Figure S3, Supporting Information).

In diabetic wounds, there is usually an increase in reactive oxygen species (ROS) and bacterial infections, leading to excessive oxidative stress, increased pro‐inflammatory cytokines, and a disrupted inflammatory response within the wound's microenvironment.^[^ ^5^, ^47^ ^]^ Therefore, the use of PC and PC‐PPCN, which possess antioxidant and antimicrobial properties that can effectively scavenge ROS free radicals and inhibit bacterial growth, could be beneficial for clinical wash solutions and regenerative dressings.

### 2.3 PC and PC‐PPCN Are Cell Compatible and Promote Dermal Fibroblast and Keratinocyte Proliferation and Migration In Vitro

Human dermal fibroblasts (HDF) and epidermal keratinocytes (HEK*α*) are two major cell types that are involved in the wound healing process and are responsible for restoring the integrity of the skin barrier.^[^ ^52^, ^53^ ^]^ In diabetes, cellular processes such as proliferation and migration of these cell types have been reported to be impaired.^[^ ^54^ ^]^ Therefore, the viability, proliferation and migration of healthy and diabetic dermal fibroblasts (HDF) and epidermal keratinocytes (HEK*α*) in response to PC and PC‐PPCN were evaluated. Initially, we assessed the cytotoxicity of PC at various concentrations to determine the suitable amount for both in vitro and in vivo studies. Various concentrations of the PC solution were added to cells and incubated at 37 °C for 24 h. The biocompatibility of the cells was evaluated using the (3‐(4,5‐dimethyl‐2‐thiazoLyl)−2,5‐diphenyl tetrazolium bromide (MTT) assay. As shown in Figure S4, Supporting Information, for PC concentrations of up to 10 mg mL^−1^, cell viability was higher than 80% after 24 h of incubation. We then assessed the cell compatibility of the test material, PC, PPCN, and PC‐PPCN, with saline used as control. The cytotoxicity of PC and PC‐PPCN to healthy and diabetic HDF and HEK*α* was as good as saline (Figure S5, Supporting Information). Therefore, it can be inferred that PC and PC‐PPCN demonstrate excellent compatibility with cells and is a safe dressing option for future in vivo applications.

**Figure 3:**
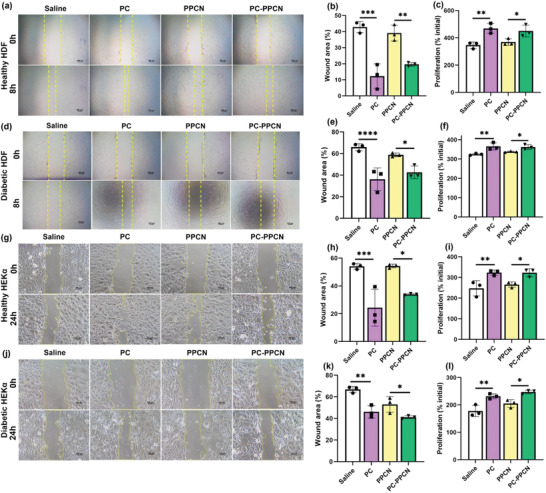
PC and PC‐PPCN promote the migration and proliferation of human dermal fibroblasts (HDF) and human epidermal keratinocytes (HEK*α*) from healthy and diabetic individuals. a) Images of healthy HDF migration after exposure to saline, PC, PPCN, and PC‐PPCN for 8 h. b) Quantification of percentage wound area remaining for healthy HDF. c) Healthy HDF proliferation at 72 h. d) Images of diabetic HDF migration after exposure to saline, PC, PPCN, and PC‐PPCN for 8 h. e) Quantification of percentage of wound area remaining for diabetic HDF. f) Diabetic HDF proliferation at 72 h. g) Images of healthy HEK*α* migration after exposure to saline, PC, PPCN, and PC‐PPCN for 24 h. h) Quantification of percentage of wound area remaining for healthy HEK*α*. i) Healthy HEK*α* proliferation at 72 h. j) Images of diabetic HEK*α* migration after exposure to saline, PC, PPCN, and PC‐PPCN for 24 h. k) Quantification of percentage of wound area remaining for diabetic HEK*α*. l) Diabetic HEK*α* proliferation at 72 h (*n* = 3; ns, not significant; *p < 0.05; **p < 0.01; ***p < 0.001, ****p < 0.0001).

To assess the efficacy of PC and PC‐PPCN in promoting cell proliferation, both healthy and diabetic HDF and HEK*α* were exposed to PC and gelled PC‐PPCN for 72 h. The number of cells was determined via PicoGreen dsDNA assay, with saline used as a control for PC, and PPCN employed as a control for PC‐PPCN. Relative to the controls, the proliferation of all cell types was significantly higher for both PC and PC‐PPCN (*p* < 0.05) (**Figure** **3**).

A scratch assay was conducted to investigate the potential of PC and PC‐PPCN to promote the migration of keratinocytes and dermal fibroblasts. Following an 8‐h incubation with PC and gelled PC‐PPCN, the percentage of wound area with both healthy and diabetic HDF showed significant reduction as compared to the saline and PPCN, respectively. Similarly, after 24 h of incubation, the percentage of wound area of both healthy and diabetic HEK*α* was significantly reduced in the presence of PC and PC‐PPCN, as compared to the saline and PPCN, respectively (*p* < 0.05) (Figure 3).

The regeneration of the epidermis and dermis relies on the proliferation and migration of both keratinocytes and dermal fibroblasts.^[^ ^55^ ^]^ By promoting the survival, proliferation, and migration of these cells, PC and PC‐PPCN could potentially accelerate wound closure rate and facilitate the formation of a stratified epithelium and granulation tissue. Consequently, the regenerated tissue quality could be further improved.

### 2.4 PC and PC‐PPCN Enhance HMVEC Migration and Proliferation and Stimulate Tubule Formation In Vitro

**Figure 4:**
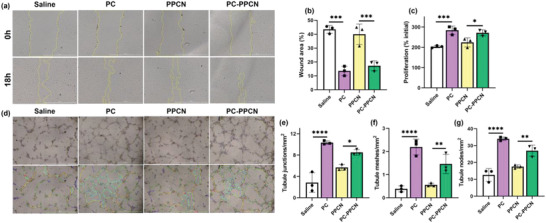
PC and PC‐PPCN promote HMVEC migration, proliferation, and tubule formation in vitro. a) Images of HMVEC migration after treatment with saline, PC, PPCN, and PC‐PPCN. b) Quantification of percentage wound area remaining on HMVEC. c) HMVEC proliferation at 72 h. d) Digital images of endothelial cell tubulogenesis after treatment with saline, PC, PPCN, and PC‐PPCN. Quantification of e) tubule junctions, f) tubule meshes, and g) tubule nodes. All data are presented as mean ± SD (*n* = 3; ns, not significant; *p < 0.05; **p < 0.01; ***p < 0.001, ****p < 0.0001).

Angiogenesis plays a significant role in wound healing by providing nutrients and oxygen to support cells and regenerated tissues.^[^ ^56^ ^]^ During this process, endothelial cells undergo a series of cellular processes including proliferation, migration, and differentiation to form new blood vessels.^[^ ^57^ ^]^ To assess the effect of PC and PC‐PPCN on endothelial cells, we performed proliferation, migration, and tubule formation assays using human dermal microvascular endothelial cells (HMVECs). Following 72 h of incubation, PC significantly stimulated endothelial cell proliferation (*p* < 0.001) when compared to the saline, while PC‐PPCN promoted a higher proliferation rate than PPCN (*p* < 0.05) (**Figure** **4**c). The migration assay of HMVEC revealed a similar trend, as both PC and PC‐PPCN significantly reduced the wound area after 18 hours of incubation when compared to saline and PPCN, respectively (*p* < 0.001) (Figure 4a,b).

Lumen formation, or tubulogenesis, is an important step during angiogenesis. To evaluate the tubulogenic capability of HMVECs treated with PC and PC‐PPCN, we conducted a tubulogenesis assay using Matrigel. The HMVECs that were exposed to PC and PC‐PPCN were seeded onto the Matrigel to examine their impact on tube formation. As illustrated in Figure 4d–g, the cells that were exposed to PC and PC‐PPCN formed a greater number of tubule junctions, nodes, and meshes than the cells that were exposed to saline and PPCN (*p* < 0.05).

Collectively, the findings confirm that PC and PC‐PPCN enhance the proliferation, migration, and tubulogenesis of dermal HMVECs, thereby potentially improving angiogenesis. As impaired angiogenesis has been shown to delay healing in diabetic wounds, the enhanced proangiogenic properties of PC and PC‐PPCN could potentially facilitate the formation of new blood vessels in the wounds, providing the necessary supply of oxygen and nutrients to the regenerating tissues and accelerating the healing process.

### 2.5 PC and PC‐PPCN Accelerate Wound Closure in a Splinted Full Thickness Excisional Wound Diabetic Mouse Model

**Figure 5:**
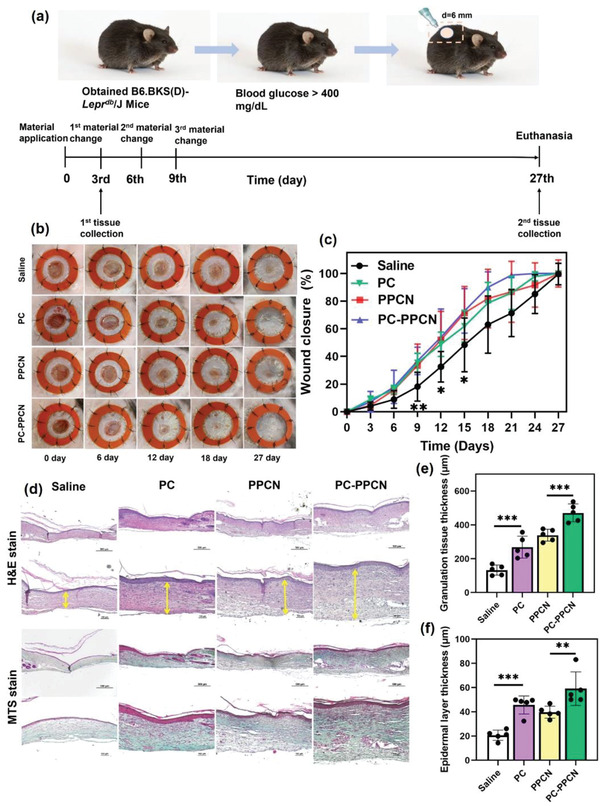
PC and PC‐PPCN accelerate wound closure and stimulate granulation tissue and keratinocyte layer formation in a diabetic mouse model. a) Experimental design and timeline of interventions. b) Digital images of the wounds after treatment with saline, PC, PPCN, and PC‐PPCN at different time points. c) Quantification of wound areas at different time points. d) Representative tissue sections from the wound sites collected on day 27 and stained with H&E and MTS. The yellow arrows indicate the thickness of granulation tissue. e) Quantification of granulation tissue thickness. f) Quantification of epidermal layer thickness. All data are presented as mean ± SD (*n* = 5; ns, not significant; *p < 0.05; **p < 0.01; ***p < 0.001, ****p < 0.0001).

Given the in vitro findings discussed in the aforementioned paragraph, we postulated that the augmented proliferation and migration of dermal and endothelial cells, along with the antioxidant and antibacterial properties and tubulogenic capacity of PC and PC‐PPCN would result in a faster wound closure response in vivo. We investigated the treatment efficacy of PC as a wound cleansing solution and PC‐PPCN as a new regenerative dressing in a splinted excisional full‐thickness wound in db/db diabetic mice. Diabetes status of the mice was confirmed throughout the study. The splinted excisional full thickness wound model was utilized to reduce contraction of mouse skin and better assess tissue regeneration. We used sterile saline, a commonly used wound cleansing solution as a reference for the PC solution, and PPCN hydrogel as the reference for PC‐PPCN. Wounds treated with PC, PC‐PPCN, and PPCN exhibited significantly faster wound closure rates (wound area of 65% at day 9) when compared to saline‐treated wounds (wound area of 82% at day 9). By day 21 after wounding, wounds treated with PC‐PPCN had completely closed, whereas those treated with saline had 30% open wound area remaining. Furthermore, the wound area of PC‐ and PPCN‐treated wounds was only 15%, as per **Figure** **5**b,c. Although there was no significant difference in the wound closure rate between PPCN‐ and PC‐PPCN‐treated wounds, there was a significant difference in the quality of the regenerated skin. These differences are discussed in Sections 2.6 and 2.8. These results demonstrate that PC and PC‐PPCN accelerate diabetic wound closure.

### 2.6 PC and PC‐PPCN Promote Granulation Tissue Formation, Re‐Epithelialization, and Neovascularization In Vivo

Regenerated skin tissue samples were collected on day 27, processed, and stained with hematoxylin and eosin (H&E) and Masson trichrome staining (MTS) for histological analysis. Granulation tissue formation in the wound beds was significantly enhanced in the PC‐ and PC‐PPCN‐treated wounds, which exhibited approximately 136 µm and 132 µm more thickness than the saline‐ and PPCN‐treated wounds, respectively (Figure 5e). The epidermal layer of the PC‐ and PC‐PPCN‐treated wounds was 34 µm and 22 µm thicker than the saline‐ and PPCN‐treated wounds, respectively (Figure 5f). The impact of PC and PC‐PPCN on granulation tissue thickness followed a similar trend for tissue samples collected on day 3 post‐wounding (Figure S13, Supporting Information).

**Figure 6:**
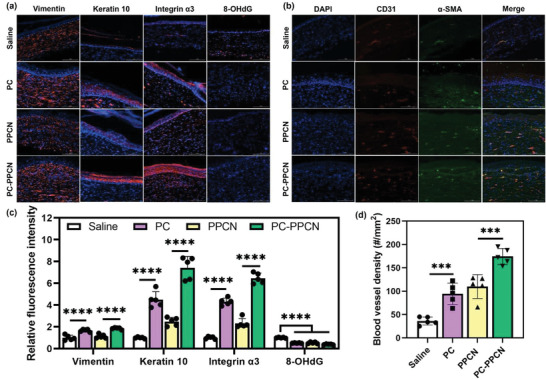
PC and PC‐PPCN promote keratinocyte and fibroblasts migration and proliferation and enhance blood vessel formation in vivo. Representative immunofluorescence images of tissues obtained at day 27 post‐wounding that were probed for: a) vimentin, keratin 10, integrin *α*3, and 8‐OHdG and b) CD31 and *α*‐smooth muscle actin (*α*‐SMA). Blue fluorescence corresponds to cell nuclei stained with 4′,6‐diamidino‐2‐phenylindole (DAPI); green fluorescence corresponds to the expression of *α*‐SMA, red fluorescence corresponds to the expression of vimentin, keratin 10, integrin *α*3, 8‐OHdG, and CD31 as indicated. c) Relative fluorescence intensity from the immunofluorescence images for vimentin, keratin 10, integrin *α*3, and 8‐OHdG at day 27 after treatment with saline, PC, PPCN, and PC‐PPCN. d) Quantification of blood vessel density for CD31 and *α*‐SMA staining. All data are presented as mean ± SD (*n* = 5; ns, not significant; *p < 0.05; **p < 0.01; ***p < 0.001, ****p < 0.0001).

Re‐epithelialization and granulation tissue formation are two vital processes in wound healing.^[^ ^56^ ^]^ Therefore, we probed tissue sections harvested on day 27 for cytokeratin 10 and vimentin, markers for spinous keratinocytes and fibroblasts, respectively. PC‐ and PC‐PPCN‐treated wounds formed a more mature spinous layer composed of K10^+^ keratinocytes compared to saline‐ and PPCN‐treated wounds, respectively (**Figure** **6**a). Moreover, PC‐ and PC‐PPCN‐treated wounds exhibited twice the vimentin expression when compared to saline‐ and PPCN‐treated wounds, respectively (Figure 6c). To further assess re‐epithelialization and granulation tissue formation, we also probed tissue sections for integrin *α*3. Previous research has demonstrated that integrin *α*3 plays a critical role in guiding keratinocyte migration, regulating epidermal growth and differentiation, and supporting both fibroblast migration and granulation tissue formation.^[^ ^58^ ^]^ PC‐ and PC‐PPCN‐treated wounds displayed upregulation of integrin *α*3 compared to saline‐ and PPCN‐treated wounds, respectively (Figure 6c). The increased granulation tissue is generally considered a positive indicator of wound healing progression. Granulation tissue provides a supportive matrix for cell migration, angiogenesis, and tissue remodeling.^[^ ^59^ ^]^

Angiogenesis is pivotal for diabetic wound healing as it provides nutrients to regenerated tissues.^[^ ^60^ ^]^ Significantly more blood vessel structures were observed in PC‐ and PC‐PPCN‐treated wounds than in saline‐ and PPCN‐treated wounds as per histology and immunofluorescence probing for CD31 and *α*‐smooth muscle actin (*α*‐SMA). Quantitative results demonstrated that the blood vessel density in the PC‐ and PC‐PPCN‐treated wounds was 2.6 and 1.6‐fold higher than saline‐ and PPCN‐treated wounds, respectively (Figure 6d).

Taken together, these results demonstrate that PC and PC‐PPCN stimulate re‐epithelialization, granulation formation, and angiogenesis in vivo. These findings suggest that the PC and PC‐PPCN treatments may lead to better quality of regenerated tissue.

### 2.7 PC and PC‐PPCN Reduce Oxidative Tissue Damage and Facilitate M1 to M2 Phenotype Macrophage Transition

Diabetic wounds are often under oxidative stress and when chronically inflamed, they fail to transition into the proliferation phase of healing.^[^ ^54^ ^]^ Due to their antioxidant properties, PC, PC‐PPCN, as well as PPCN were expected to scavenge ROS, thus reducing oxidative stress and inhibiting DNA damage in the wound environment. Oxidized DNA in the wound was evaluated by probing for 8‐OHdG. The wounds treated with PC, PPCN, and PC‐PPCN had a significant reduction in oxidized DNA when compared to saline‐treated wounds (Figure 6a,c).

**Figure 7:**
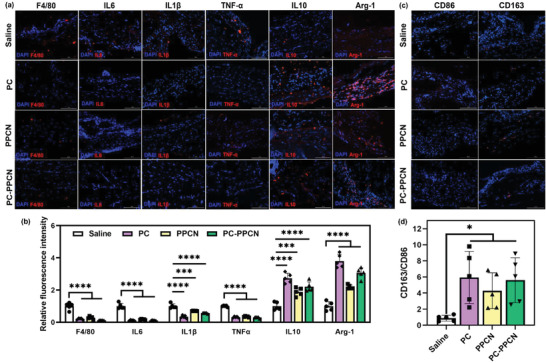
PC and PC‐PPCN modulate inflammatory responses and facilitate the transition from M1 to M2 macrophage phenotype in vivo. a) Representative immunofluorescence images of tissues sampled at 3 days post‐wounding and probed for F4/80, IL6, IL1*β*, TNF‐*α*, IL10, and Arg‐1. Blue fluorescence corresponds to cell nuclei stained with 4′,6‐diamidino‐2‐phenylindole (DAPI); red fluorescence corresponds to the expression of F4/80, IL6, IL1*β*, TNF‐*α*, IL10, and Arg‐1 as indicated. b) Relative fluorescence intensity from the immunofluorescence images in panel (a). c) Representative immunofluorescence images of tissues sampled at 3 days post‐wounding and probed for CD86 and CD163. d) Quantification of the ratio of CD163 and CD86. All data are presented as mean ± SD (*n* = 5; ns, not significant; *p < 0.05; **p < 0.01; ***p < 0.001, ****p < 0.0001).

The excessive ROS in the diabetic wound environment leads to chronic inflammation and increased expression of pro‐inflammatory cytokines, such as IL‐6, IL‐1*β*, and TNF‐*α* by M1 macrophages as well as a significant reduction in anti‐inflammatory M2 phenotype macrophages that express anti‐inflammatory cytokines including IL‐10 and Arg‐1.^[^ ^61^ ^]^ Therefore, a wound dressing that can scavenge ROS is able to modulate inflammatory response in the wound environment. To further investigate the effect of PC and PC‐PPCN on modulating inflammatory response in the wound, the tissues were collected on day 3 post‐wounding and the inflammatory cells and their secreted cytokines were evaluated by immunofluorescence. The F4/80^+^ inflammatory cell density in PC‐ and PC‐PPCN‐ as well as PPCN‐treated wounds was significantly reduced when compared to the saline‐treated wounds (*p* < 0.0001) (**Figure** **7**a). However, the staining of F4/80 in the tissue collected at day 27 revealed very few positive cells (Figure S14, Supporting Information).

Both pro‐inflammatory (IL‐6, IL‐1*β*, and TNF‐*α*) and anti‐inflammatory cytokines (IL‐10 and Arg‐1) expression in diabetic wounds were assessed as well (Figure 7a). Compared to the saline‐treated wounds, a decrease in various pro‐inflammatory cytokines and a significant increase in anti‐inflammatory cytokines was observed in PC‐, PC‐PPCN‐ as well as PPCN‐treated wounds (*p* < 0.0001) (Figure 7b). To further confirm the impact of PC and PC‐PPCN on inflammation modulation in vivo, immunofluorescence probing of CD86 and CD163, two markers for M1 and M2 phenotype macrophages, respectively, was performed. When compared to the wounds treated with saline, a significant decrease in the number of CD86^+^ cells was observed in the wounds treated with PC, PC‐PPCN, as well as PPCN (*p* < 0.0001). The density of CD163^+^ cells did not differ among all groups (Figure S19, Supporting Information). However, when considering the CD163/CD86 ratio, the wounds treated with PC, PC‐PPCN, as well as PPCN showed a significantly higher ratio than the saline‐treated wounds (*p* < 0.05) (Figure 7d). These results suggest a polarization of macrophages toward the M2 phenotype. Therefore, PC and PC‐PPCN modulate the inflammatory response in the early wound healing process and facilitate the transition from inflammatory M1 to reparative M2 macrophage phenotype.

In summary, macroscopic and microscopic observations from the in vivo study suggest that the use of PC as a wash solution and PC‐PPCN as a regenerative wound dressing exerts a positive impact on various stages of diabetic wound healing, thus promoting the overall healing process.

### 2.8 PC and PC‐PPCN Improve the Mechanical and Electrophysiological Properties of Regenerated Tissue

**Figure 8:**
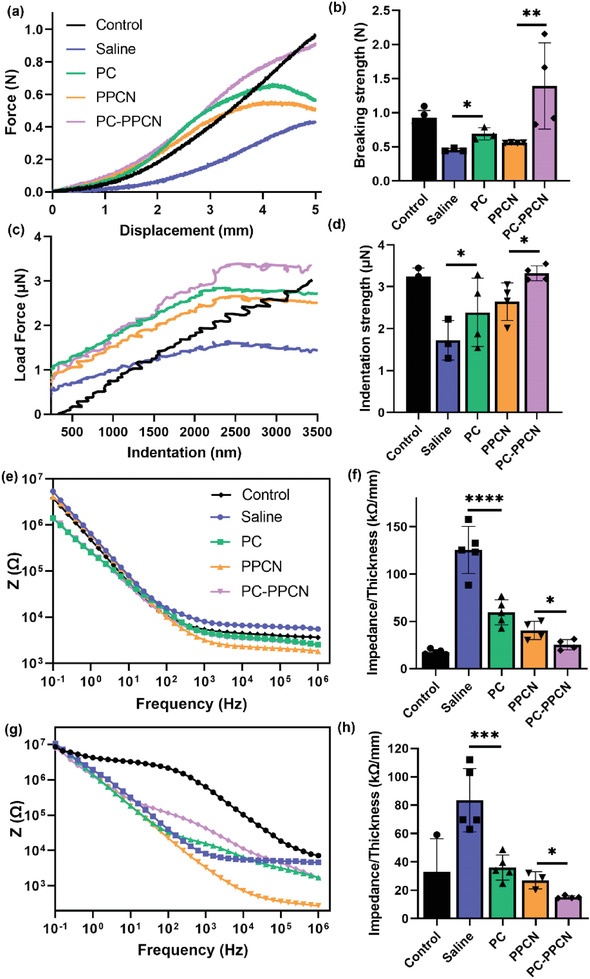
PC and PC‐PPCN improve mechanical and electrical properties of regenerated tissue. a) Tensile test of regenerated skin treated with saline, PC, PPCN, and PC‐PPCN. Uninjured db/db mouse skin was used as control. b) Breaking strength reached with 5 mm displacement. c) Indentation measurement of regenerated skin. d) Indentation strength achieved with 10 µm indentation. e) Electrochemical impedance spectra of regenerated skin in the direct configuration. f) The direct impedance of the regenerated skin at 0.1 Hz. g) Bode plots of the regenerated skin in the capacitive configuration. h) The capacitive impedance of the regenerated skin at 0.1 Hz. All data are presented as mean ± SD (*n* = 5; ns, not significant; *p < 0.05; **p < 0.01; ***p < 0.001, ****p < 0.0001).

Regenerated tissue quality refers to the characteristics of newly formed tissue that has regenerated in response to an injury or trauma.^[^ ^62^ ^]^ The quality of regenerated tissue can be evaluated by various parameters, such as mechanical strength and electrical properties.^[^ ^63^ ^]^ The regenerated tissue quality is an important determinant of the overall success of the healing process and functional recovery of the injured tissue. The assessment of regenerated tissue quality is essential to evaluate the efficacy of various treatment strategies and to guide clinical decision‐making in wound care management.^[^ ^63^ ^]^ To further evaluate regenerated tissue quality, we investigated their mechanical and electrical properties. A rectangular skin sample with the healed wound at the center was subjected to a tensile test. The skin was stretched at a rate of 5 mm min^−1^ until a displacement of 5 mm was achieved. Uninjured skin from db/db mice was utilized as the control. The force‐displacement curve for all the regenerated skin samples exhibited a similar pattern, with a drop in force at some point, while the force for the uninjured skin continued to increase (**Figure** **8**a). The breaking strength of PC and PC‐PPCN treated wounds was significantly enhanced compared to saline and PPCN‐treated wounds, respectively (*p* < 0.05). Of note, the PC‐PPCN‐treated wounds displayed higher breaking strength than uninjured skin (Figure 8b). Indentation measurements were also performed on the regenerated skin to determine the maximum indentation force that the skin could withstand. Similar to the tensile test results, the force–indentation curve displayed a comparable pattern with a force drop at some point, while the force for the uninjured skin continued to increase (Figure 8c). The indentation strength of PC‐ and PC‐PPCN‐treated wounds was significantly higher when compared to saline‐ and PPCN‐treated wounds, respectively (*p* < 0.05). Similar to tensile test results, the PC‐PPCN‐treated wounds displayed higher breaking strength than uninjured skin (Figure 8d). The thickness of the skin is one of the main factors that determines its mechanical properties.^[^ ^63^, ^64^ ^]^ The outcomes of both tensile and indentation tests are consistent with the findings from histological analysis. The increased breaking strength can potentially be attributed to the formation of well‐organized and robust granulation tissue, which includes new blood vessels, fibroblasts, and collagen deposition.^[^ ^65^ ^]^ These results suggest improved tissue regeneration and strength rather than fibrosis.^[^ ^66^ ^]^ Furthermore, over time, as remodeling goes to completion, mechanical properties may approach those of normal tissue.

The electrophysiological balance of skin is a critical regulator of skin health and repair.^[^ ^67^ ^]^ The application of bioelectrical impedance analysis can provide clinicians with a novel technique for monitoring cellular changes and identifying potential complications during wound healing.^[^ ^68^, ^69^ ^]^ We measured the impedance of regenerated tissue via electrochemical impedance spectroscopy (EIS) in both capacitive and direct configurations. For capacitive measurements, one electrode contacted the epidermis, and one contacted the dermis. In the direct configuration, both electrodes were in contact with the dermis. Impedance values were compared at 0.1 Hz. In both configurations measured, impedance of PC‐ and PC‐PPCN‐treated wounds was significantly lower than saline‐ and PPCN‐treated wounds, respectively (*p* < 0.05) (Figure 8e–h). Furthermore, impedance was lowest for the PC‐PPCN‐treated wounds, which was similar to uninjured skin. The reduced impedance may signal bolstered skin hydration, improved collagen alignment, or enhanced skin barrier integrity, which are collectively indicative of improved tissue quality.^[^ ^70^, ^71^ ^]^

Patients with peripheral neuropathy are susceptible to developing diabetic foot ulcers (DFU), which are often caused by repetitive stress on an area experiencing high vertical or shear stress. DFU recurrence is frequently observed, with 40% of patients experiencing a wound recurrence within a year of the ulcer healing.^[^ ^3^ ^]^ One of the most common physical factors associated with recurrence of diabetic foot ulcer is a weakened healed skin.^[^ ^3^, ^72^ ^]^ As a result, there is a significant need for wound treatments that not only accelerate wound closure but also improve the quality of regenerated tissue, in order to enhance the outcomes of wound care management for individuals with diabetes.

A wound wash solution is a sterile solution that is used to clean and irrigate wounds. It is typically used to remove dirt, debris, and other contaminants from the wound and to help prevent infection. The role of wash solutions in wound care management is important.^[^ ^73^ ^]^ Hydrogen peroxide solution is a commonly used wound cleansing agent, but it has been found to impair re‐epithelialization and decrease wound strength.^[^ ^74^ ^]^ Compared to sterile saline, the most commonly used wound cleansing solution, a PC solution is able to scavenge free radical and inhibit bacterial growth according to our findings. It also stimulates re‐epithelialization and granulation tissue formation, resulting in healed skin with significantly enhanced mechanical strength and electrophysiological properties when compared to saline. When utilized in a regenerative dressing, PC‐PPCN has demonstrated the ability to restore the mechanical and electrophysiological properties of regenerated skin to levels that are similar to those of uninjured skin.

## 3 Conclusion

**Figure 9:**
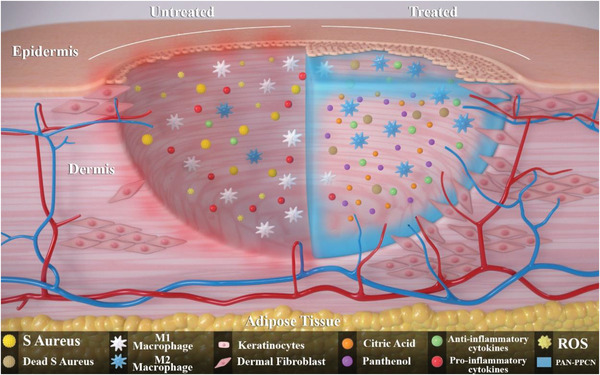
Illustration of the potential mechanisms for PC and PC‐PPCN to accelerate diabetic wound closure, including facilitating macrophage polarization from M1 to M2, inhibiting bacterial infection, promoting skin cell migration, and stimulating angiogenesis in the db/db mouse model.

We synthesized the novel compound panthenol citrate from the condensation reaction of citric acid with the provitamin B panthenol with properties that are beneficial to tissue regeneration in diabetes. PC is a versatile compound as it can be used either as a wash/cleansing solution or wound dressing to tackle impaired wound healing in diabetes. The antioxidant, antibacterial, anti‐inflammatory, and proangiogenic properties of PC lead to an acceleration of wound closure and improvement of regenerated tissue quality as mechanical and electrophysiological properties are restored (**Figure** **9**). Both in vitro and in vivo data presented support the potential use of PC in skin tissue engineering as well as other clinical applications that require these properties. Future studies will investigate the use of PC and PC‐PPCN for wound healing in a large animal model.

## 4 Experimental Section

### Materials

d,l‐panthenol, citric acid, polyethylene glycol (PEG 400), glycerol 1,3‐diglycerolate diacrylate (GDD), and calcium pantothenate were purchased from Millipore Sigma (Burlington, MA). *N*‐Isopropylacrylamide (NIPAAM) was purchased from Thermo Fisher Scientific (Haverhill, MA). Human dermal fibroblasts (HDF) and human keratinocyte cells (HEK*α*) that were obtained from both healthy and diabetic individuals and their culture medium were purchased from Zen‐Bio (Durham, NC). Human primary umbilical vein endothelial cells (HMVEC) and all the cell culture media and their growth kits were purchased from Lonza Bioscience (Cambridge, MA).

### Synthesis of Panthenol Citrate (PC)

Equimolar amounts of d,l‐panthenol (20 g, 0.1 mole), and citric acid (18.7 g, 0.1 mole) (1:1) were added to a 250 mL round‐bottom flask and heated up to 120 °C with a nitrogen purge. After melting, the mixture reacted at this temperature for 2 h. The resulting solution was cooled down to room temperature, dissolved in ethanol, and precipitated in acetone. After vacuuming dry, the yellowish honey‐like liquid was obtained with a yield of 73%.

### Synthesis of Panthenol Citrate PPCN (PC‐PPCN)

Panthenol citrate poly(polyethylene glycol citrate) acrylate (PC‐PPCac) prepolymers with a molar ratio of 1:5:8:1 were synthesized by combining panthenol, citric acid, polyethylene glycol, and glycerol 1,3‐diglycerolate diacrylate and heated up to 120 °C with a nitrogen purge. After melting, the mixture reacted at this temperature for 2 h. Water‐soluble thermoresponsive photoluminescent hydrogels were synthesized via free radical polymerization by reacting PC‐PPCac with NIPAAm. Briefly, equivalent amounts of PC‐PPCac and NIPAAm were added into a three‐neck round‐bottom flask and reacted in 1,4‐dioxane at 65 °C for 16 h in a nitrogen atmosphere, using AIBN (6.5 × 10^−3^ m in concentration) as an initiator. The obtained poly (panthenol citrate polyethylene glycol citrate co‐*N*‐isopropylacrylamide) (PC‐PPCN) copolymer was diluted in 1,4‐dioxane and purified by precipitation in diethyl ether and vacuum dried. PPCN was synthesized as previously described.^[^ ^40^ ^]^ Briefly, a polycondensation reaction at 140 °C for 45 min was used to make poly(polyethylene glycol citrate) acrylate prepolymer (PPCac) by melting citric acid, PEG, and glycerol 1,3‐diglycerolate diacrylate in a 5:9:1 molar ratio under constant stirring. In a three‐necked flask, PPCac and NIPAAm were mixed in a 1:1 mass ratio and dissolved in 1,4‐dioxane. The PPCac and NIPAAm mixture was treated with Azobis(isobutyronitrile) (AIBN) radical initiator and reacted for 16 h at 65 °C in a nitrogen atmosphere. The reaction products were then dissolved in 1,4‐dioxane and vacuum‐dried after precipitation in diethyl ether. All the materials were neutralized to pH 7.4 by 1 m NaOH solution. PPCN and PC‐PPCN were dialyzed with 2 kDa molecular weight‐cutoff (MWCO) dialysis tubing for 2 days at 4 °C. Then all the materials were lyophilized for 2 days.

### Characterization of PC and PC‐PPCN

^1^H NMR spectrum of PC, PC‐PPCN, and PPCN was recorded using an Ag500 NMR spectrometer (Bruker, Billerica, MA) at ambient temperature, using DMSO‐d_6_ as solvent and tetramethylsilane (TMS) as an internal reference. FID resolution was 0.158 Hz/point, corresponding to a sweep width of 10.3 kHz and an acquisition time of 3.07 s. The molar ratios of panthenol, citric acid, PEG, GDD, and NIPAAm in PC‐PPCN were determined to be 1.2:1.6:2.1:1:14.3 from ^1^H NMR. The amount of PC in PC‐PPCN was 10.6 mol%. Attenuated total reflection‐Fourier transform infrared (ATR‐FTIR) spectra were obtained from Thermo Nicolet Nexus 870 spectrometer equipped with a Pike horizontal ATR accessory with a covered sample trough, using 8 cm^−1^ spectral resolution and accumulation of 64 scans, the reflectance element was a ZnSe crystal with ten internal reflections. Ultraviolet absorption spectra were recorded using an Agilent Cary 100 UV/Vis spectrophotometer (Agilent, Senta Clara, CA) from 200 to 700 nm. The photoluminescent properties of the PC were investigated on a PC1 photon counting spectrofluorometer (ISS, Champaign, IL). The slit widths of both excitation and emission were set at 0.5 nm for all samples except where otherwise indicated. The fluorescent intensity of the PC in an aqueous solution (10 mg mL^−1^) was recorded at 450 nm for emission and excited at 350 nm. The molecular weight of PC was measured by electrospray ionization mass spectroscopy (ESI) using Agilent 1200 series high performance liquid chromatography (HPLC) connected to an Agilent 6210A time‐of‐flight (TOF) mass spectrometer. Samples were prepared by dissolving 1 mg mL^−1^ PC in methanol. A 10 µL injection of each sample was eluted using 80% methanol and 20% dichloromethane as mobile phase through a C18 trap column with a flow rate of 3 mL min^−1^. The molecular weight of PPCN was characterized by Tosoh EcoSEC size exclusion chromatography System (GPC) equipped with a Tosoh Super AW3000 column and a Tosoh SperAW4000+ guard column. 1 mg mL^−1^ PPCN was dissolved and eluted with dimethylformamide (DMF) and 0.01 m lithium bromide (LiBr). The results were detected by a refractive index (RI) detector and analyzed using polyethylene oxide (PEO) calibration standards. Dynamic light scattering (DLS) was used to detect the hydrodynamic diameter of PPCN and PC‐PPCN nanoparticles. 1 mL of 1 mg mL^−1^ PPCN and PC‐PPCN were first dissolved tetrahydrofuran (THF) and added into 10 mL water dropwise while stirring. The resulting solution was stirred for 2 h to allow the THF evaporation. The hydrodynamic diameter of the nanoparticles was obtained using Nanodrop DLS (Unchained labs, Pleasanton, CA). Mettler Toledo TGA/DSC 3+ simultaneous thermal analyzer (Mettler Toledo, Columbus, OH) was used to determine the thermal stability of PC‐PPCN and PPCN materials. The sample was heated from 50 to 500 °C with a heating rate of 10 °C min^−1^ under a continuous nitrogen flow of 50 mL min^−1^. A DSC 250 (TA Instrument, New Castle, DE) was used to determine the thermal transition behavior of PC‐PPCN and PPCN. The samples were heated from −50 to 150 °C with a heating rate of 10 °C min^−1^ and a continuous nitrogen flow of 50 mL min^−1^.

### Phase Transition and Viscoelastic Properties of PC‐PPCN

The viscoelastic properties of neutralized PPCN and PC‐PPCN hydrogels with 100 mg mL^−1^ in PBS have been studied in a Discovery Hybrid DHR‐3 (TA, New Castle, DE) rheometer. The analyses were conducted at a frequency of 1.5 Hz and a heating rate of 2 °C min^−1^, using a 1.5% strain and a 50 rad s^−1^ angular frequency, in the temperature range from 15 up to 45 °C. Storage modulus (*G*′) and loss modulus (*G*″) changes of PPCN and PC‐PPCN solutions were recorded.

### Antioxidant Assays

The ability of different materials to scavenge the free radical cation, 2,2′‐azino‐bis(3‐ethylbenzthiazoline‐6‐sulfonic acid) (ABTS) was assessed. A stock solution of 7 mm ABTS and 2.45 mm sodium persulfate in MQ water was prepared and left for 16 h in the dark at room temperature, after which the solution was sequentially filtered by 0.45 um filters. This working solution was then exposed to samples and incubated at 37 °C. At each time point (2, 8, 24, and 48 h), ABTS solution was sampled, and diluted with MQ water 1:1, and the absorbance was measured at 734 nm. All measurements were performed in triplicate. The free radical scavenging activity was measured as % inhibition of free radicals by measuring the decrease in absorbance compared to control solutions.

(2) $$\mathrm{Proliferation}\;\mathrm{percentage}\;=\frac{\mathrm{Fluorescence}\;\mathrm{intensity}\;\mathrm{after}\;72\;h\;\mathrm{incubation}-\mathrm{Fluorescence}\;\mathrm{intensity}\;\mathrm{before}\;\mathrm{adding}\;\mathrm{materials}}{\mathrm{Fluorescence}\;\mathrm{intensity}\;\mathrm{before}\;\mathrm{adding}\;\mathrm{materials}}\;\times 100\%$$

The antioxidant activity of polymers was also evaluated using the *β*‐carotene‐linoleic acid bleaching assay with a few modifications as described below. Briefly, tween 40 (4 g), *β*‐carotene (4 mg), and 0.5 mL linoleic acid were mixed in 20 mL chloroform. After removing chloroform in a rotary evaporator, 30 mL of pre‐warmed Britton buffer (100 mm, pH 6.5) was added to 1 mL of the oily residue with vigorous stirring. Aliquots (1 mL) of the obtained emulsion were added to the samples. Reaction mixtures were incubated at 45 °C for a different time point (10, 30, 60, and 100 min). Spontaneous oxidation of linoleic acid at 45 °C led to *β*‐carotene discoloration, which was monitored by the decrease in absorbance at 470 nm, starting immediately after sample preparation (*t* = 0 min).

The iron chelation activity was assessed by incubating samples with 0.25 mm FeCl_2_·4H_2_O solution (50 mg mL^−1^) at 37 °C. Supernatants were collected at each time point (0, 5, 15, and 30 min) and reacted with 5 mm ferrozine indicator solution in a 5:1 ratio. Ferrous ions chelated by polymers will not be available for ferrozine reaction, resulting in lower color development. Absorbance was measured at 534 nm and the percentage of iron chelated was calculated. For all the antioxidant assays, ePTFE, saline, and panthenol were used as negative controls, and citric acid and l‐ascorbic acid were used as positive controls.

### Antimicrobial Assay

*S. aureus* was cultured in a Luria–Bertani (LB) broth at 200 rpm and 37 °C overnight. 100 µL of PC, PPCN, PC‐PPCN, panthenol, citric acid, and calcium pantothenate solution were added into a 48‐well plate and mixed with 100 µL of bacteria suspension (in PBS, 1.5 × 10^4^ CFU mL^−1^). Saline was used as a control. Subsequently, the 48‐well plate was put into an incubator at 37 °C for 24 h. Then the bacteria suspension was diluted with 800 µL of sterilized PBS. 20 µL of the collected bacteria suspension was spread on an LB agar plate (15 mL of LB agar solution in a 10 mm Petri dish). After overnight incubation, the digital images of each agar plate were taken and the colony‐forming units (CFU) on the Petri dish were counted using ImageJ software. Each group was tested in triplicate and the killing ratios of bacteria were calculated by the following equation:

(1) $$\begin{array}{c} \mathrm{killing}\;\mathrm{ratio}\;=\frac{\mathrm{CFU}\;\mathrm{of}\;\mathrm{control}\;\mathrm{group}-\mathrm{CFU}\;\mathrm{of}\;\mathrm{treated}\;\mathrm{group}}{\mathrm{CFU}\;\mathrm{of}\;\mathrm{control}\;\mathrm{group}}\;\times 100\% \end{array}$$

### In Vitro Cell Viability Evaluation of PC and PC‐PPCN

Healthy and diabetic adult human epidermal keratinocyte (HEK*α*) (Zenbio, passage 1–2) and human dermal fibroblast (HDF) (Zenbio, passage 1–2) were cultured in keratinocyte and fibroblast growth medium, respectively, in a humidified incubator equilibrated with 5% CO_2_ at 37 °C. The cell viability of PC at different concentrations was measured to determine the concentration of PC used for both in vitro and in vivo studies. Cells were seeded at a density of 1 × 10^4^ cells per well in 96‐well tissue culture plates. After overnight incubation, 100 µL of PC solution at different concentrations (0.1, 1, 2, 5, 10, 20 mg mL^−1^) were added into each well and incubated for 24 h. Then the cell viability of PC was measured by MTT assay.

For cell compatibility assessment of PC and PC‐PPCN, cells were seeded in 24‐well tissue culture plate at a density of 5 × 10^4^ cells per well and incubated at 37 °C overnight. The cells were treated with PC solution (10 mg mL^−1^ in saline) and gelled PPCN and PC‐PPCN hydrogels (100 mg mL^−1^ in saline). Cell viability was assessed after 24 h of culture using alamarBlue assay.

### In Vitro Cell Proliferation Evaluation of PC and PC‐PPCN

Healthy and diabetic HEK*α* and HDF (Zen‐bio, passage 1–2), and HMVEC (Lonza,

passage 1–2) proliferation assays were conducted by seeding cells at a density of 5 × 10^4^ cells per well in 24‐well tissue culture plates and incubated overnight to allow cell attachment. The cells were treated with 10 mg mL^−1^ of PC solution and 100 mg mL^−1^ of gelled PC‐PPCN and PPCN hydrogels. Extracts of PPCN and PC‐PPCN gels were prepared by immerging 100 µL of 100 mg mL^−1^ PC‐PPCN and PPCN hydrogels in 1 mL cell culture medium, respectively, and incubated 24 h at 37 °C. After 72 h of incubation, the cell quantity was measured by PicoGreen dsDNA assay, and the proliferation rate was calculated by the equation below.

The diabetic keratinocytes used in this study were directly purchased from a company that isolated cells from the skin of diabetic patients. Although diabetic cells may show hyperproliferation in vivo, their behavior in vitro can be different due to intrinsic characteristics of the cells or culture conditions.^[^ ^75^, ^76^, ^77^ ^]^ In our in vitro experiments, similar to findings by others, we observed that diabetic keratinocytes exhibited relatively lower proliferation rates compared to healthy cells.

### In Vitro Cell Migration Evaluation of PC and PC‐PPCN

Scratch assays using healthy and diabetic HEK*α* and HDF (Zen‐bio, passage 1–2), and HMVEC (Lonza, passage 1–2) were performed by creating a wound via scraping on the cell monolayer. Cells were incubated at 37 °C with saline, PC solution, PPCN, and PC‐PPCN hydrogels. Digital images were taken at different time points. The percentage of wound area was calculated by the equation below.

(3) $$\mathrm{Wound}\;\mathrm{area}\;\left(\%\right)\;=\frac{A\left(t\right)\;}{A\left(0\right)}\;\times 100\%$$

where *A*(*t*) is the wound area at time *t* and *A*(0) is its initial area.

### Endothelial Tubule Formation Assay

HMVECs (passage 3) were seeded in 24‐well plates (5 × 10^4^ cells per well) and incubated overnight. Cells were then incubated with saline, PC solution, PPCN, and PC‐PPCN hydrogels for 24 h, respectively. Matrigel (BD Biosciences, San Jose, CA, USA) was diluted twofold with vascular basal medium, added to a 96‐well plate (60 µL per well), and preincubated at 37 °C for 30 min. The cells were then collected and plated on the 96‐well plate (2.5 × 10^4^ cells per well) and incubated for 4 h. The enclosed networks of complete tubes were digitally imaged and quantified using the Angiogenesis Analyzer plugin on ImageJ.

### In Vivo Wound Healing Experiment of PC and PC‐PPCN

Male, 8–12‐week‐old db/db mice (BKS.Cg‐Dock7m +/+ Leprdb/J Homozygous for Leprdb) were purchased from the Jackson Laboratory. Mice were housed in the Center for Comparative Medicine at Northwestern University. All animal protocols were approved by Northwestern University's Institutional Animal Care and Use Committee with approval number IS00000373. The in vivo performance of the PC and PC‐PPCN was evaluated using a splinted excisional wound model in db/db mice whose blood glucose was continuously above 400 mg dL^−1^ for a week (Figure S12, Supporting Information). All the animals exhibited normal activity levels and regular eating patterns. Over the course of 30 days, they gradually experienced a 10% weight loss, which was consistent with previous reports and probably due to the high blood glucose concentration.^[^ ^78^ ^]^ There was no other therapeutic used during the experiments. Mice were separated into six groups of five animals, with each group receiving one of the following treatments: 1) PC, 2) PPCN, 3) PC‐ PPCN, and saline as control. To prevent skin contraction, paired sterilized doughnut‐shaped silicon rubber splints (10‐mm inner diameter; 12‐mm outer diameter) were attached to the left and right dorsal sides of the mouse with interrupted 6‐0 nylon sutures (Ethicon, Cincinnati, OH) after depilation. A 6‐mm circular, full‐thickness wound was made in the center of each splinted area. 40 µL of 10 mg mL^−1^ of PC solution, and 100 mg mL^−1^ of PPCN and PC‐PPCN hydrogel were applied to each wound bed. A transparent sterile occlusive dressing TegaDerm (3 m, Saint Paul, MN) was then placed over the wound and the splint. The wound dressings were changed three times (day 3, day 6, and day 9 post‐wounding) and then left covered with TegaDerm. Then, the TegaDerm was changed every 3 days until euthanasia at day 27). Digital images of the wound area were taken every three days and quantified using ImageJ by normalizing the wound area to the known splint area at each time point.

### Tissue Processing

After 3 days post‐wounding and upon full closure of the wounds (27 days), animals were euthanized, and the regenerated wound tissue was excised with a 10‐mm biopsy punch (Acuderm, Fort Lauderdale, FL), fixed using 4% paraformaldehyde overnight, washed with PBS twice every 2 h and then overnight. After fixation, the tissues were dehydrated in gradually ascending ethanol solutions (70%, 80%, 95%, and 100% twice) for 45 min each time. The tissues were then cleared in xylene for 1 h twice, and paraffin overnight at 60 °C. On the next day, the tissues were transferred to an embedding machine and immersed in paraffin overnight, and then embedded in a paraffin mold.

### Histological and Immunohistochemical Analysis

The tissues were sectioned 5‐µm thickness onto microscope slides. Tissue sections were then stained for hematoxylin and eosin (H&E) and Masson's trichrome staining (MTS) on the tissues collected on day 27. The tissue sections were also immunofluorescently stained by fibroblast marker, Vimentin, keratinocyte marker, Cytokeratin‐10, cell integrin marker, Integrin *α*3, endothelial cell marker, CD31, and *α*‐Smooth muscle actin (*α*‐SMA), and DNA damage marker, 8‐OHdG. The inflammation markers were stained on tissue collected on day 3 using F4/80, CD86 and CD163 macrophage, pro‐inflammatory cytokines, IL‐6, IL1*β* and TNF‐*α*, anti‐inflammatory cytokines, IL‐10 and Arg‐1. Slides sections were deparaffinized, and rehydrated tissue sections were immersed in pH 6 citrate buffer to perform the heat‐induced epitope retrieval. After cooling down to room temperature, tissue sections were rinsed with PBS three times and blocked with 5 mg mL^−1^ bovine serum albumin solution for 30 min. Primary antibodies IgG against Vimentin (1:200 clone EPR3776; catalog No. ab92547, Abcam), Cytokeratin‐10 (1:50 clone RKSE60; catalog No. sc‐23877, Santa Cruz Biotechnology), Integrin *α*3 (1:50 clone E‐8; catalog No. sc‐393298, Santa Cruz Biotechnology), CD31 (1:200 clone EPR17259; catalog No. ab182981, Abcam), *α*‐Smooth muscle actin (1:1000 clone 4A4; catalog No. ab119952, Abcam), F4/80 (1:50 clone M‐300; catalog No. sc‐25830, Santa Cruz Biotechnology), CD86 (1:200 clone B7‐2; catalog No. 14‐0862‐82, Invitrogen), CD163 (1:200 clone EPR19518; catalog No.ab182422, Abcam), IL‐6 (1:50; catalog No. ab208113), IL1*β* (1:100; catalog No. B600‐633, Novusbio), TNF‐*α* (1:50 clone 52B83; catalog No. ab1793, Abcam), IL10 (1:50; catalog No. ab9969, Abcam) and Arg‐1 (1:50 clone E‐2; catalog No. sc‐271430, Santa Cruz Biotechnology), were applied on tissue sections and incubated overnight at 4 °C. After rinsing with PBS for 5 min three times, the fluorescently labelled secondary antibodies, Alexa Fluor 594 anti‐mouse (1:1000; catalogue No. A11032; Invitrogen) or Alexa Fluor 488 anti‐mouse (1:1000; catalogue No. A11001; Invitrogen) or Alexa Fluor 594 anti‐rabbit (1:1000; catalogue No. A 32754; Invitrogen) were applied and incubated for 30 min at room temperature.  The tissue sections were then rinsed with PBST buffer for 5 minutes six times. Nuclear counterstain (4,6‐diamidino‐2‐phenylindole (DAPI)) was then applied and the coverslips were mounted. All stained sections were imaged using Cytation 5 cell imaging multimode reader and the fluorescence intensity was quantified by ImageJ software. At least five images for each group were used for the quantification. The blood vessel density was quantified by specifically counting cells in the lumen doubled stained for CD31 and SMA. Three sections per animal were used for all the quantification.

### Tensile and Indentation Test

The animals were euthanized 27 days post‐wounding, and the resulting regenerated wound tissue was collected and trimmed to a rectangular shape measuring 2 cm in length and 1 cm in width. Tensile tests were then conducted on the wound tissue using an Instron 5544 mechanical tester that had a 500 N load cell (Instron, Canton, MA). During the tests, the rectangular sample was pulled at a rate of 5 mm min^−1^ until it reached a displacement of 5 mm, and the maximum force was recorded. Each group consisted of five samples that were measured. Additionally, indentation tests were performed on the wound tissue using a Piuma nanoindenter (Optics11 Life, Boston, MA). For these tests, the samples were immersed in a thin layer of PBS within a petri dish, and a 10 µm indentation was applied to each sample, with the maximum force recorded.

### EIS Measurement

Electrochemical impedance spectroscopy (EIS) was performed using a PalmSens 7 and data was collected using PSTrace 5.9 software. A custom apparatus was designed to secure the skin during EIS measurements (Figure S20, Supporting Information). Skin was placed between two identical custom‐designed parts with electrode pins (Digikey 952–2601) mounted in the center slots. The apparatus was secured with nylon screws to ensure that skin and electrode pins did not move during the experiment. For EIS, frequency varied between 0.1 and 10^6^ Hz with a potential of 0.01 V. Direct measurements were taken 6 mm apart, spanning across the wound, with pins directly in contact with the hypodermis. For capacitive measurements, pins were lined up on either side of the skin. After testing, impedance was taken at 0.1 Hz to compare.

### Statistical Analysis

All data are expressed as mean ± standard deviation (SD) (*n* = 3 for all the in vitro studies, and *n* = 5 for all the in vivo studies). All experimental data were analyzed for statistical significance by one‐way ANOVA with Bonferroni multiple comparison corrections. Statistical analyses were performed using GraphPad Prism 9 software; *p < 0.05, **p < 0.01, ***p < 0.001, and ****p < 0.0001.

## Conflict of Interest

G.A.A. is an inventor on patents that disclose PC and PC‐PPCN. The remaining authors declare no conflict of interest.

## Supporting information

Supporting Information

Supplemental Video 1

## Data Availability

The data that support the findings of this study are available from the corresponding author upon reasonable request.
