# Supplementary material for: Panthenol Citrate Biomaterials Accelerate Wound Healing and Restore Tissue Integrity
Source: Adv Healthc Mater. 2023 Jun 25;12(31):2301683. doi: 10.1002/adhm.202301683 (PMC11468745; doi:10.1002/adhm.202301683)
Supplement: Supplementary file 1 — Supporting Information [file ADHM-12-2301683-s002.pdf]

ADVANCED HEALTHCARE MATERIALS

Supporting Information

for *Adv. Healthcare Mater.*, DOI 10.1002/adhm.202301683

Panthenol Citrate Biomaterials Accelerate Wound Healing and Restore Tissue Integrity

*Huifeng Wang, Chongwen Duan, Rebecca L. Keate and Guillermo A. Ameer**

Supporting information

Panthenol Citrate Biomaterials Accelerate Wound Healing and Restore Tissue Integrity

Huifeng Wang[1,2], Chongwen Duan[1], Rebecca L. Keate[1], Guillermo A. Ameer*[1,2,3,4,5,6,7]

[1]Department of Biomedical Engineering, Northwestern University, Evanston, IL 60208, USA

[2]Center for Advanced Regenerative Engineering, Northwestern University, Evanston, IL 60208, USA

[3]Department of Surgery, Feinberg School of Medicine, Northwestern University, Chicago, IL 60611, USA.

[4]Interdisciplinary Biological Sciences, Northwestern University, Evanston, IL 60208, USA.

[5]Chemistry of Life Processes Institute, Northwestern University, Evanston, IL 60208, USA.

[6]Simpson Querrey Institute, Northwestern University, Chicago, IL 60611, USA.

[7]International Institute for Nanotechnology, Northwestern University, Evanston, IL 60208, USA.

Corresponding author: Dr. Guillermo A. Ameer Email: g-ameer@northwestern.edu

Results

To discern the individual components' impact on cell proliferation, migration, panthenol, citric acid, calcium pantothenate (vitamin B_5), and extracts or released products acquired from PPCN, and PC-PPCN (RP-PPCN and RP-PC-PPCN) were administered to the cells. The cell proliferation rates of all cell types significantly increased in response to panthenol, calcium pantothenate, and extracts obtained from PC-PPCN, compared to citric acid and extracts from PPCN (**Figure S6-S9**). Treatment with panthenol, calcium pantothenate, and extracts from PC-PPCN resulted in a decreased wound area for both keratinocytes and dermal fibroblasts compared to citric acid and extracts from PPCN (**Figure S6-S9**). For HMVECs, the use of panthenol, calcium pantothenate, and extracts from PC-PPCN resulted in an elevated proliferation rate, a decrease in wound area and a larger amount of tubule formation in comparison to citric acid and extracts from PPCN (**Figure S10-S11**).

For *in vivo* studies, the wounds treated with panthenol, and citric acid did not result in an accelerated wound closure when compared to the use of saline (**Figure S15**). However, wounds treated with panthenol exhibited a granulation tissue that was 184 µm thicker and an epidermal layer that was 24 µm thicker than that of wounds treated with saline. There was no significant difference between citric acid- and saline-treated wounds (**Figure S15**). The wounds treated with panthenol and citric acid also had increased vimentin and integrin α3 expression, while panthenol-treated wounds had increased cytokeratin 10 expression (**Figure S16**). Additionally, panthenol- and citric acid-treated wounds upregulated CD31 and α-SMA expression (**Figure S17**). There was no significant difference in macrophage or secreted cytokine expression found among panthenol-, citric acid- and saline-treated wounds (**Figure S18**).

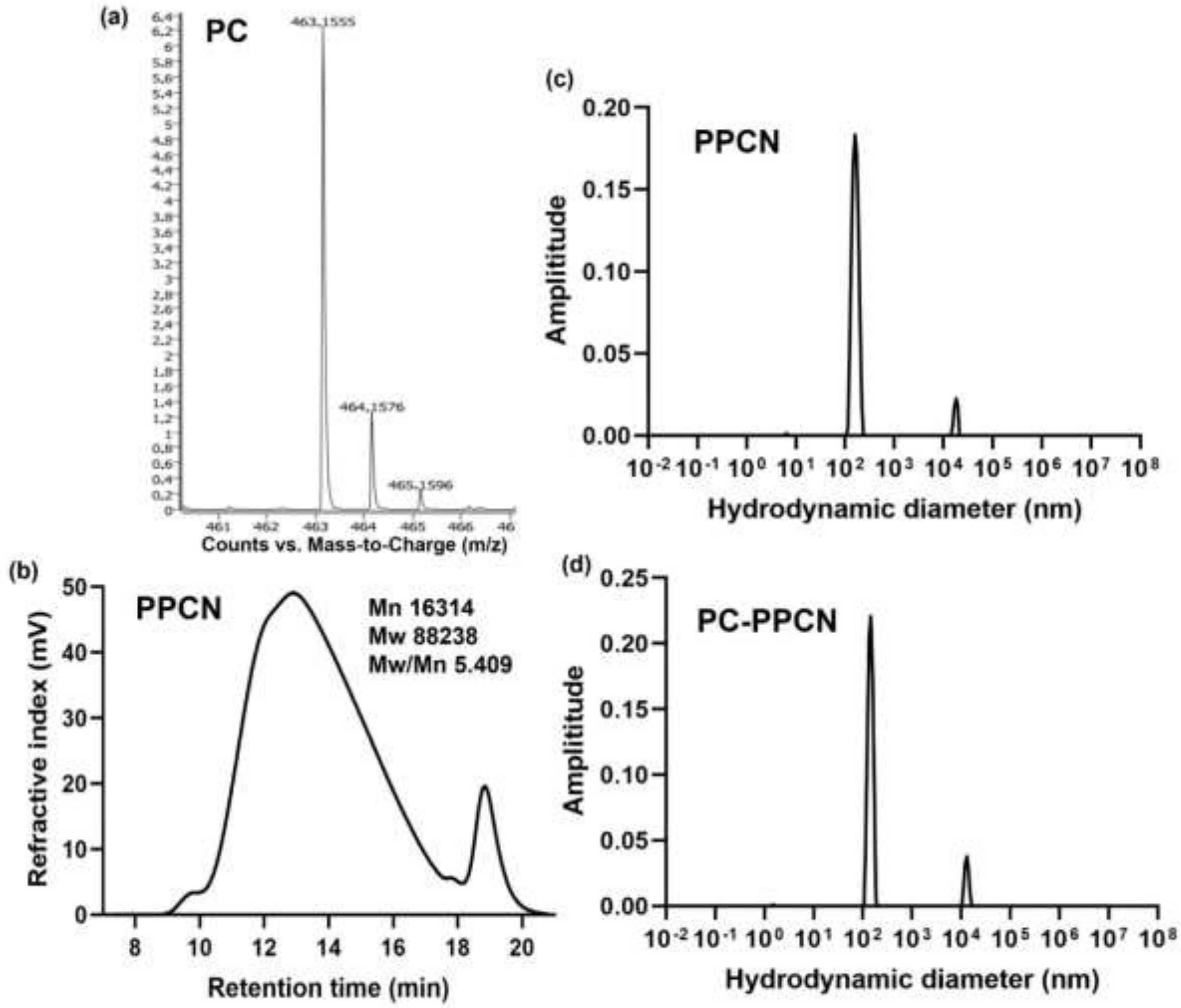

Figure S1. Molecular weight of PC and PPCN, and hydrodynamic diameter of PPCN and PC-PPCN. (a) Mass spectrum of PC. (b) GPC spectrum of PPCN. (c) and (d) DLS measurements of PPCN and PC-PPCN.

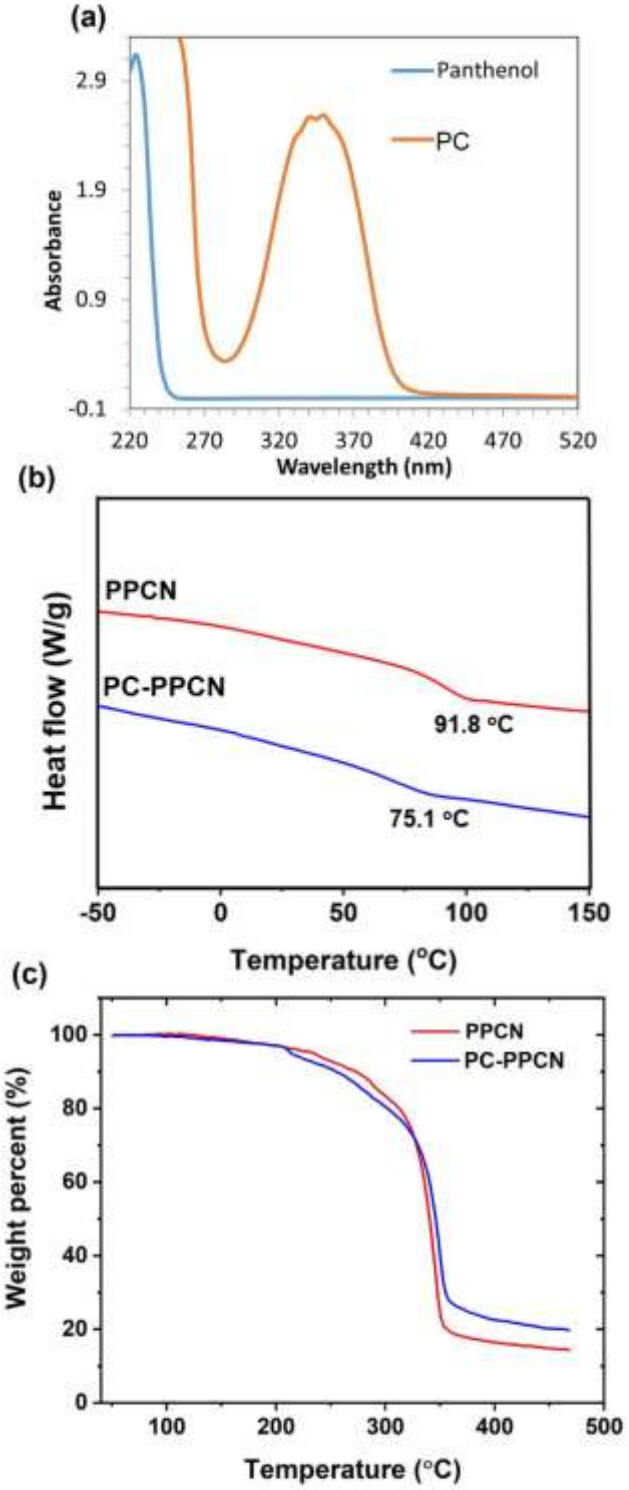

Figure S2. Optical and thermal properties of PC, PPCN, PC-PPCN. (a) UV absorbance spectrum of PC and panthenol. (b) DSC curve of PPCN and PC-PPCN. (c) TGA analysis of PPCN and PC-PPCN.

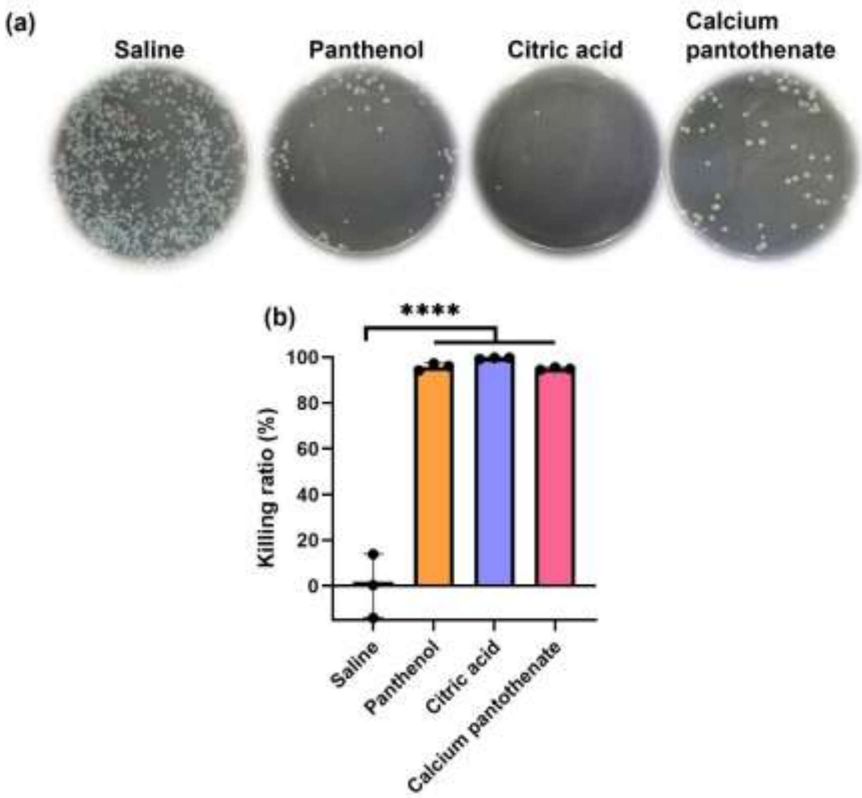

Figure S3. (a) Digital images of *S. aureus* colonies on culture plates after incubation with saline, panthenol, citric acid, and calcium pantothenate, respectively. (h) Corresponding bacteria killing ratio against *S. aureus*. All data are presented as mean ± SD (n=9 for panthenol release study and n =3 for antioxidant assays; ns, not significant; *P < 0.05; **P < 0.01; ***P < 0.001, ****P<0.0001).

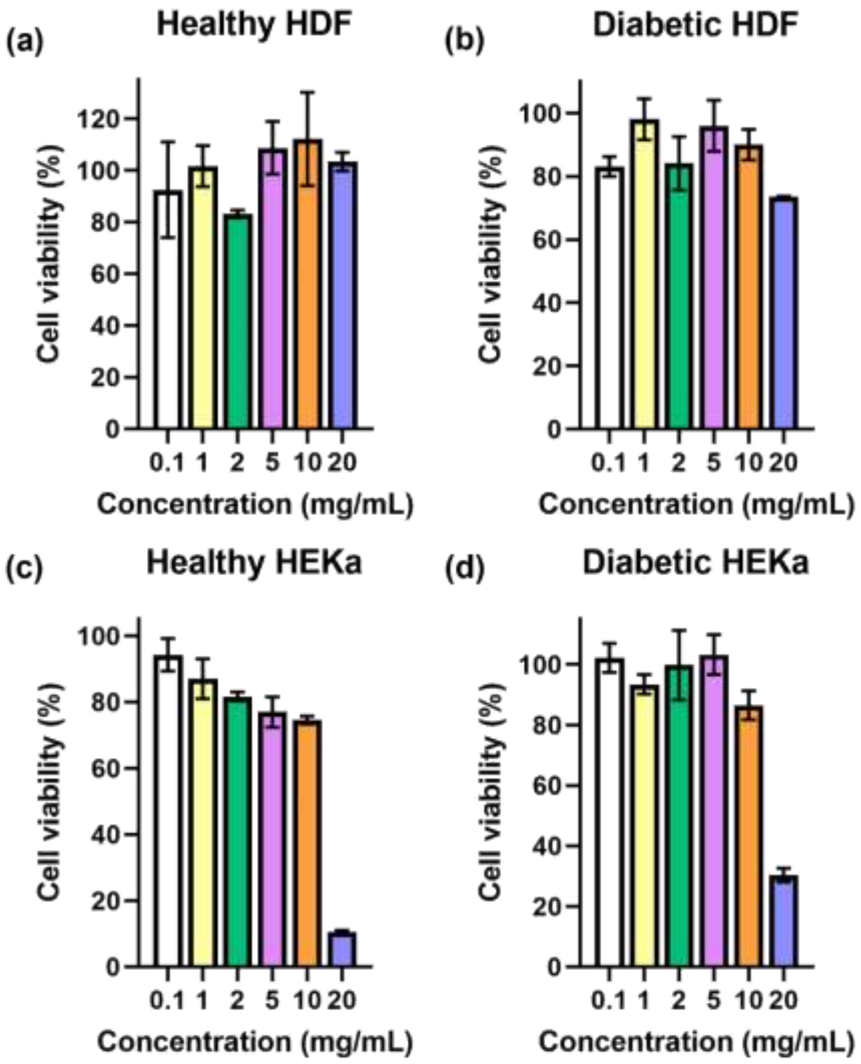

Figure S4. Cytotoxicity studies of PC with different concentrations on (a) healthy HDF (b) diabetic HDF (c) healthy HEKα, and (d) diabetic HEKα. The cell viability was normalized with the non-treated group, TCPS. All data are presented as mean ± SD (n =3; ns, not significant; *P < 0.05; **P < 0.01; ***P < 0.001, ****P<0.0001).

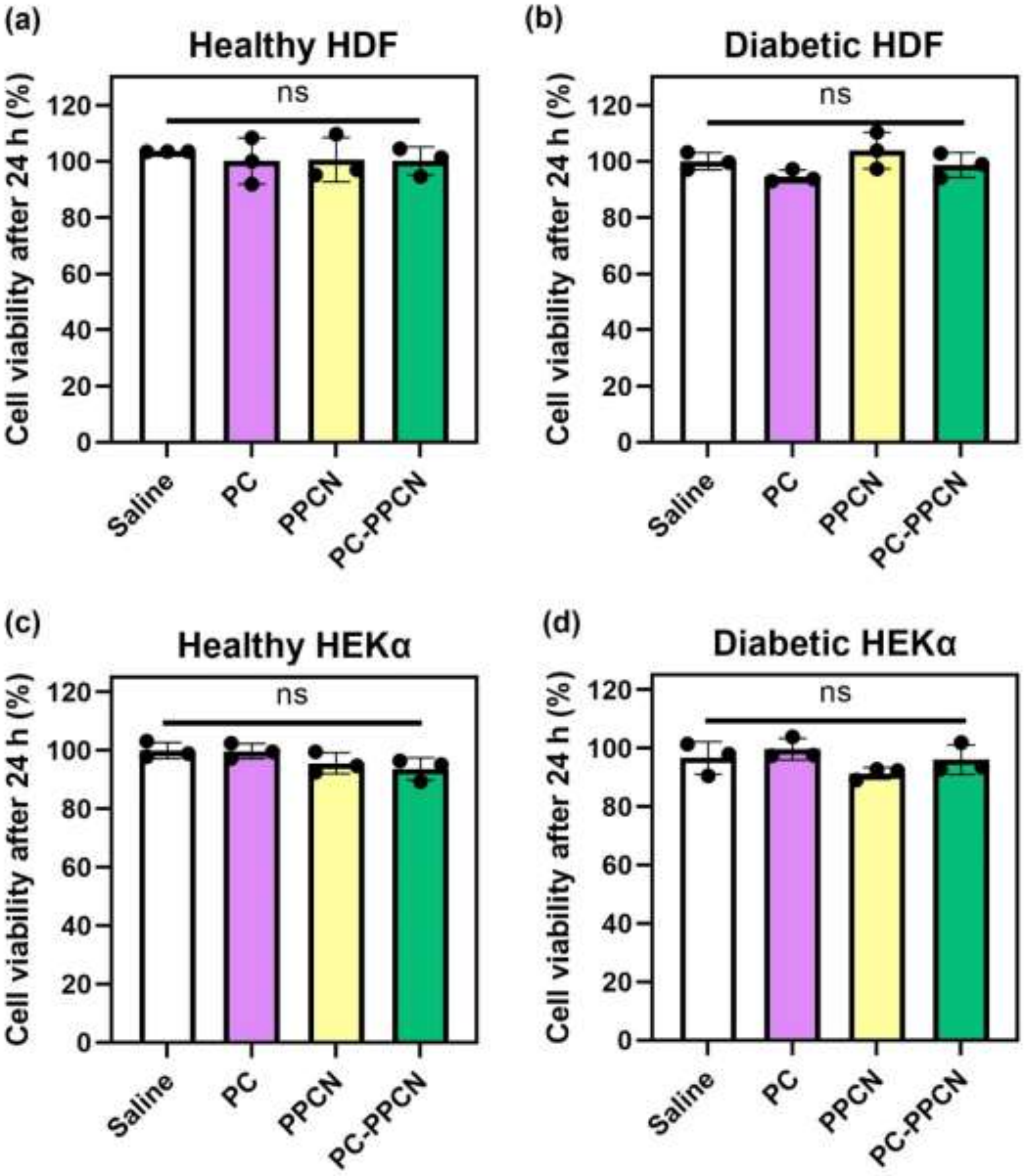

Figure S5. Cytotoxicity studies of PC, PPCN and PC-PPCN on (a) healthy HDF (b) diabetic HDF (c) healthy HEKα, and (d) diabetic HEKα. The cell viability was normalized with the non-treated group, TCPS. All data are presented as mean ± SD (n =3; ns, not significant; *P < 0.05; **P < 0.01; ***P < 0.001, ****P<0.0001).

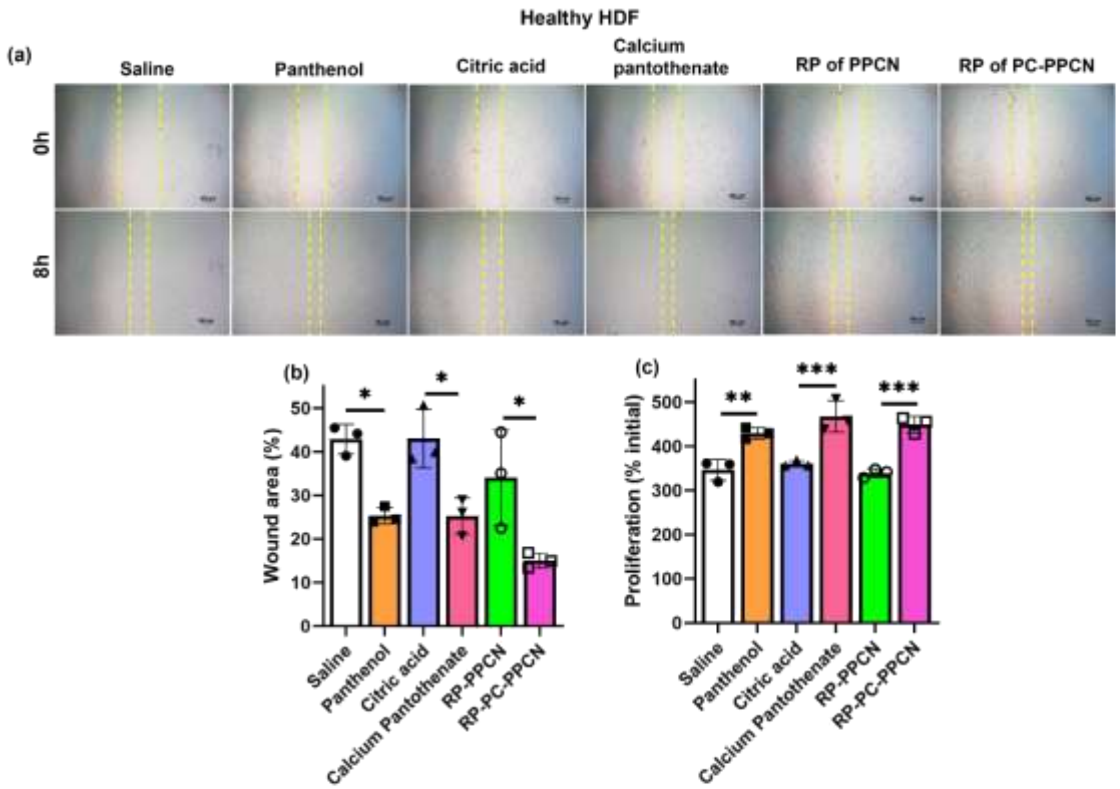

Figure S6. (a) Images of healthy HDF migration after exposure to panthenol, citric acid, calcium pantothenate, and released products from PPCN and PC-PPCN for 8 hours. (b) Quantification of healthy HDF migration. (c) Healthy HDF proliferation at 72 hours. All data are presented as mean ± SD (n =3; ns, not significant; *P < 0.05; **P < 0.01; ***P < 0.001, ****P<0.0001).

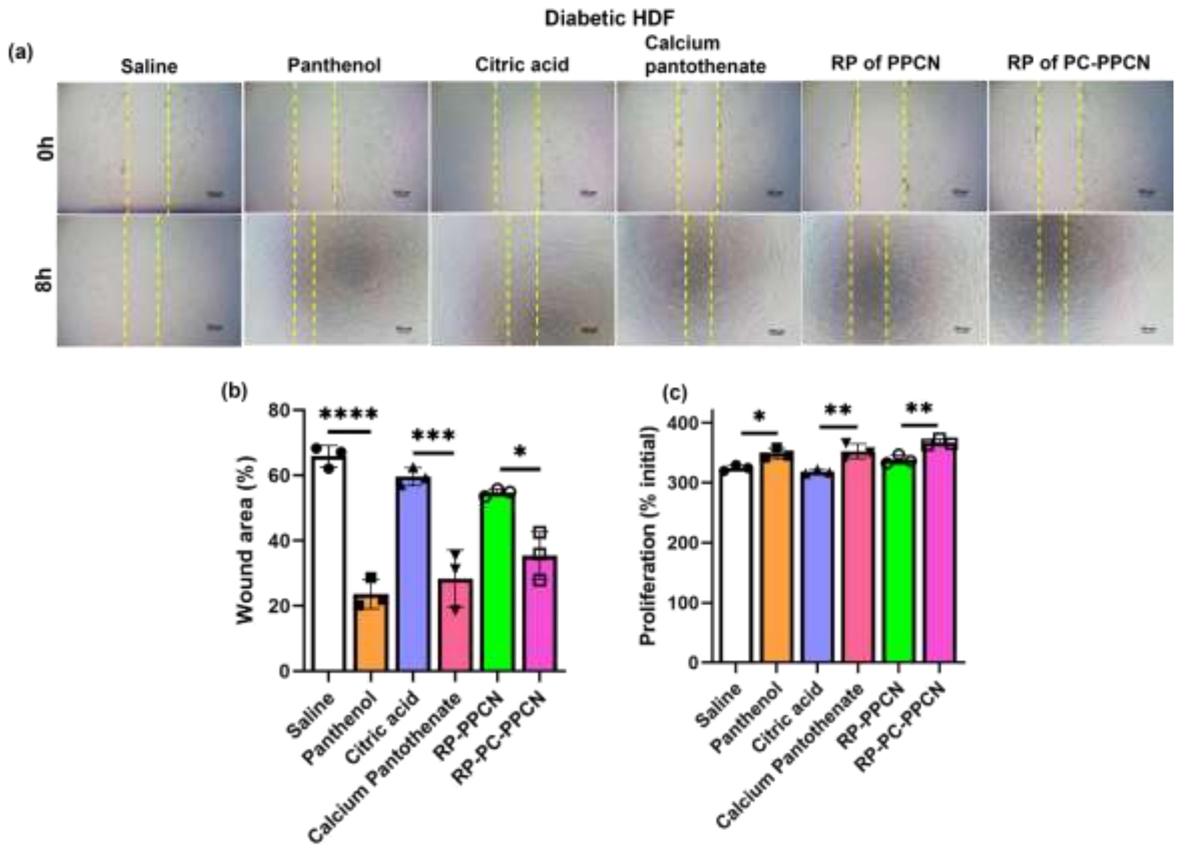

Figure S7. (a) Images of diabetic HDF migration after exposure to panthenol, citric acid, calcium pantothenate, and released products from PPCN and PC-PPCN for 8 hours. (b) Quantification of diabetic HDF migration. (c) Diabetic HDF proliferation at 72 hours. All data are presented as mean ± SD (n =3; ns, not significant; *P < 0.05; **P < 0.01; ***P < 0.001, ****P<0.0001).

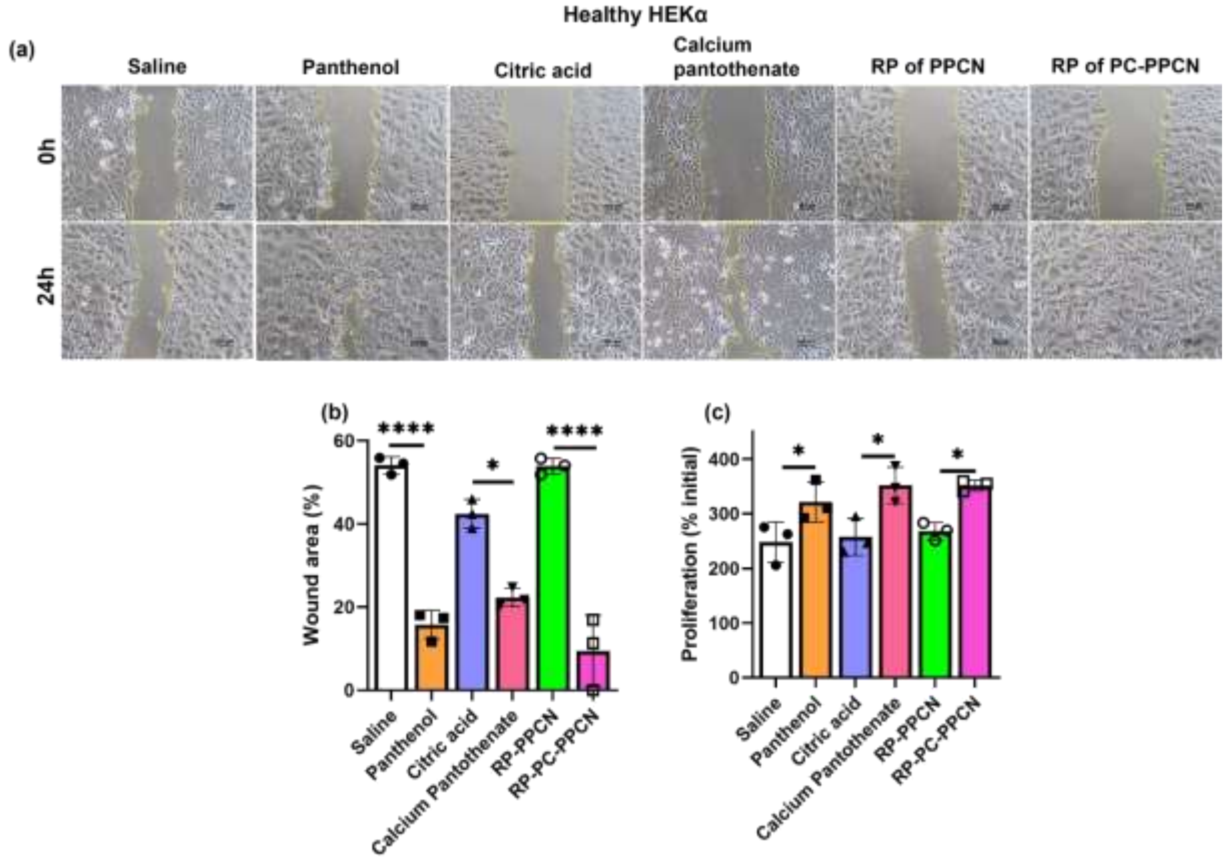

Figure S8. (a) Images of healthy HEKα migration after exposure to panthenol, citric acid, calcium pantothenate, and released products from PPCN and PC-PPCN for 24 hours. (b) Quantification of healthy HEKα migration. (c) Healthy HEKα proliferation at 72 hours. All data are presented as mean ± SD (n =3; ns, not significant; *$P < 0.05$; **$P < 0.01$; ***$P < 0.001$, ****$P<0.0001$).

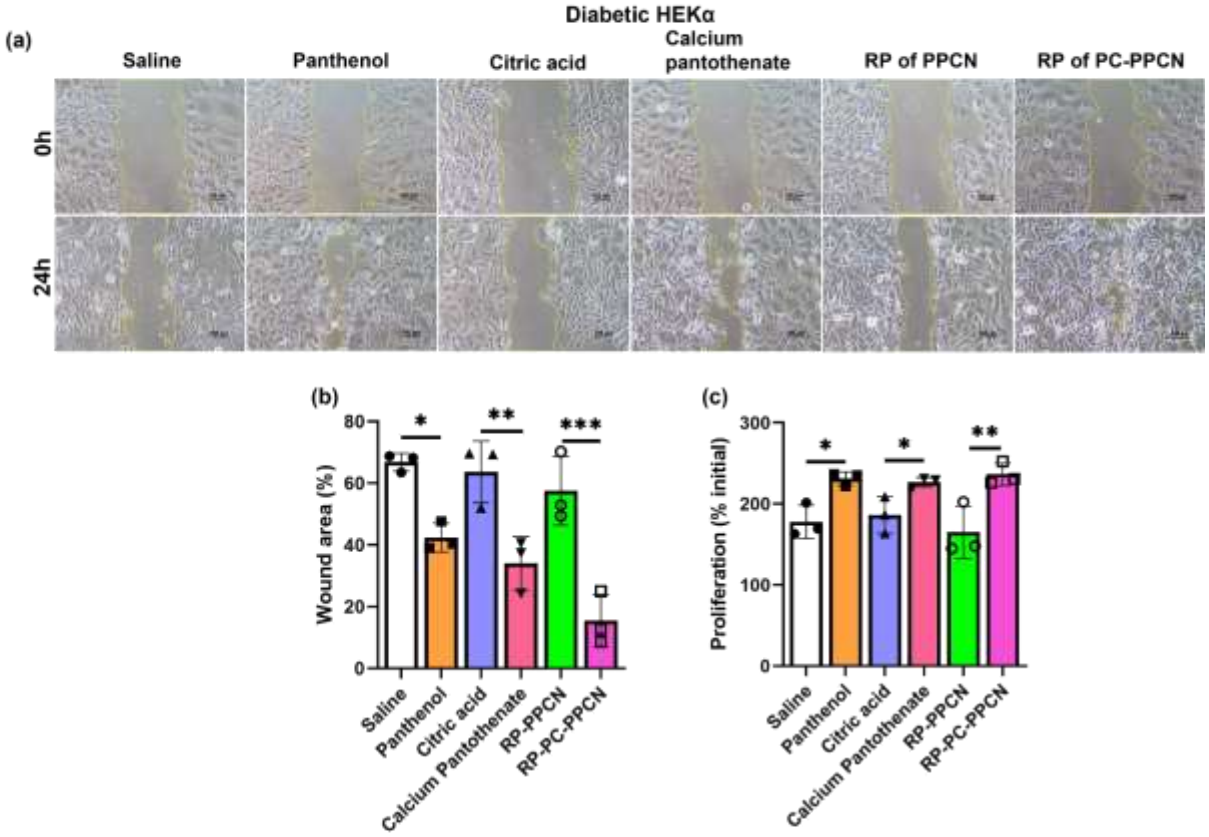

Figure S9. (a) Images of diabetic HEKα migration after exposure to panthenol, citric acid, calcium pantothenate, and released products from PPCN and PC-PPCN for 24 hours. (b) Quantification of diabetic HEKα migration. (c) Diabetic HEKα proliferation at 72 hours. All data are presented as mean ± SD (n =3; ns, not significant; *P < 0.05; **P < 0.01; ***P < 0.001, ****P<0.0001).

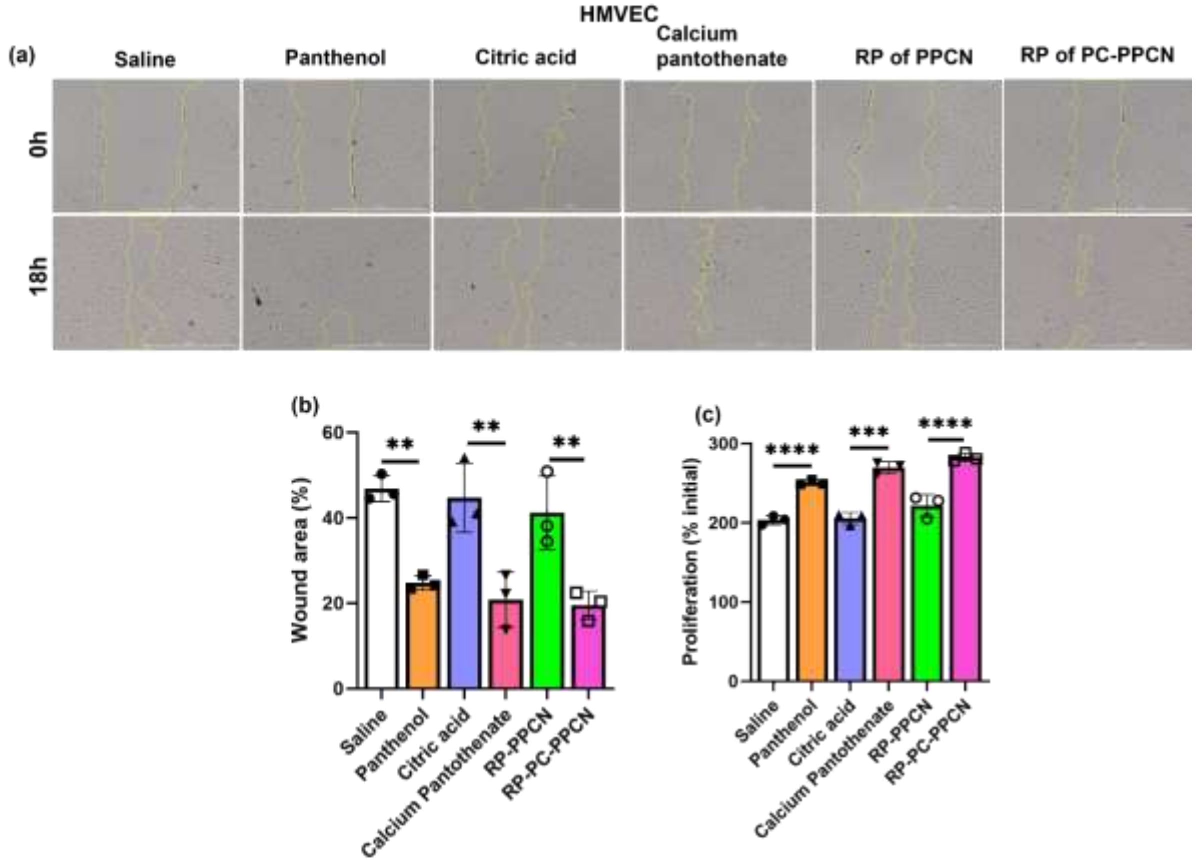

Figure S10. (a) Images of HMVEC migration after exposure to panthenol, citric acid, calcium pantothenate, and released products from PPCN and PC-PPCN for 8 hours. (b) Quantification of HMVEC migration. (c) HMVEC proliferation at 72 hours. All data are presented as mean ± SD (n =3; ns, not significant; *P < 0.05; **P < 0.01; ***P < 0.001, ****P<0.0001).

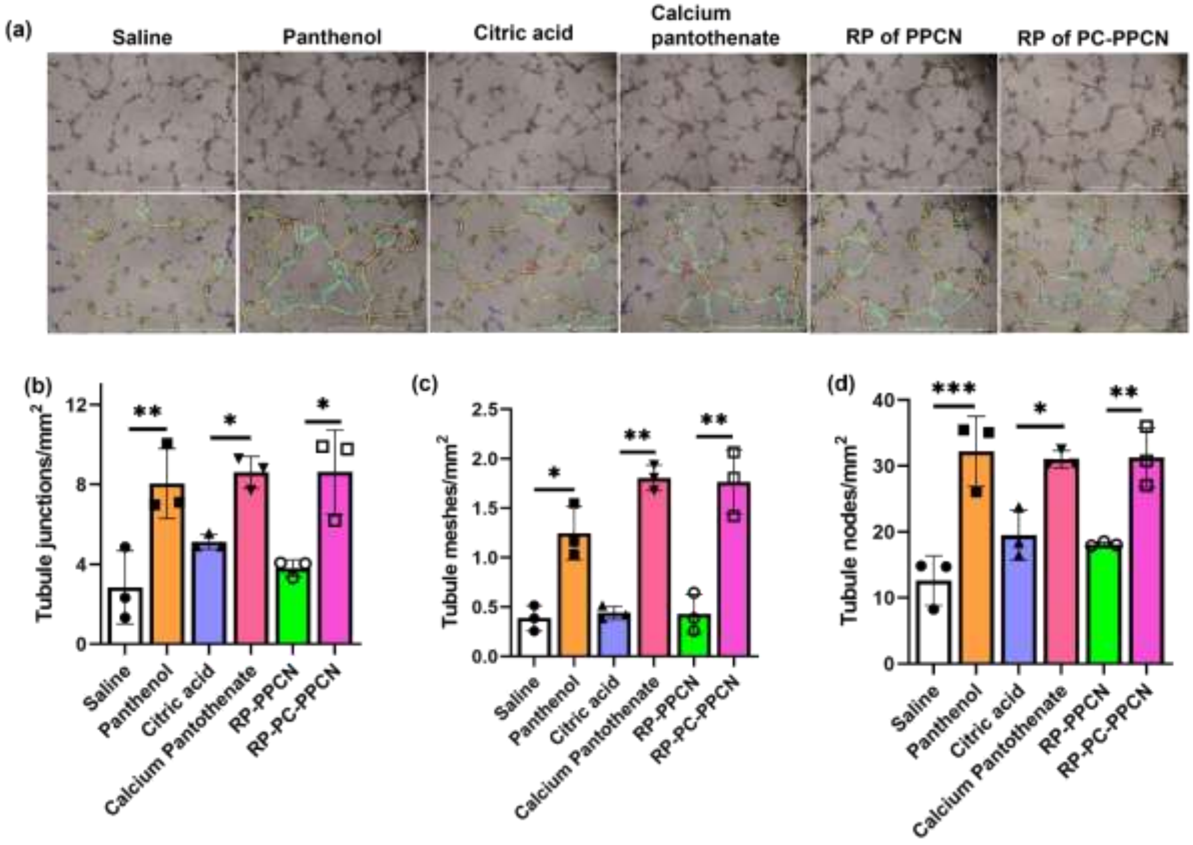

Figure S11. (a) Digital images of endothelial tubulogenesis when treated with panthenol, citric acid, calcium pantothenate, and released products from PPCN and PC-PPCN. Quantification of (b) tubule junctions, (c) tubule meshes, and (d) tubule nodes. All data are presented as mean ± SD (n =3; ns, not significant; *P < 0.05; **P < 0.01; ***P < 0.001, ****P<0.0001).

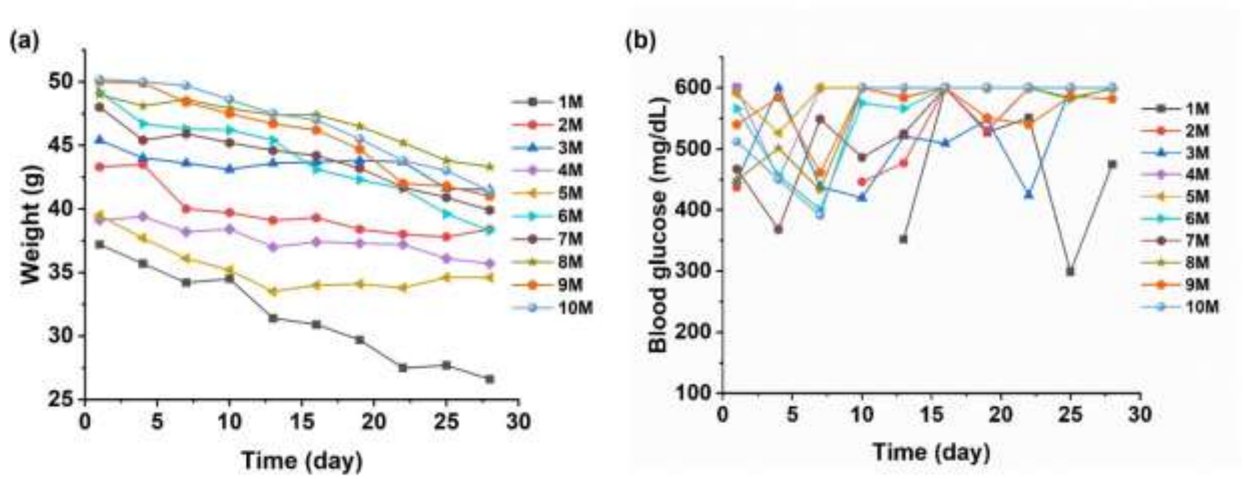

Figure S12. (a) Weight and (b) blood glucose measurement of db/db mice.

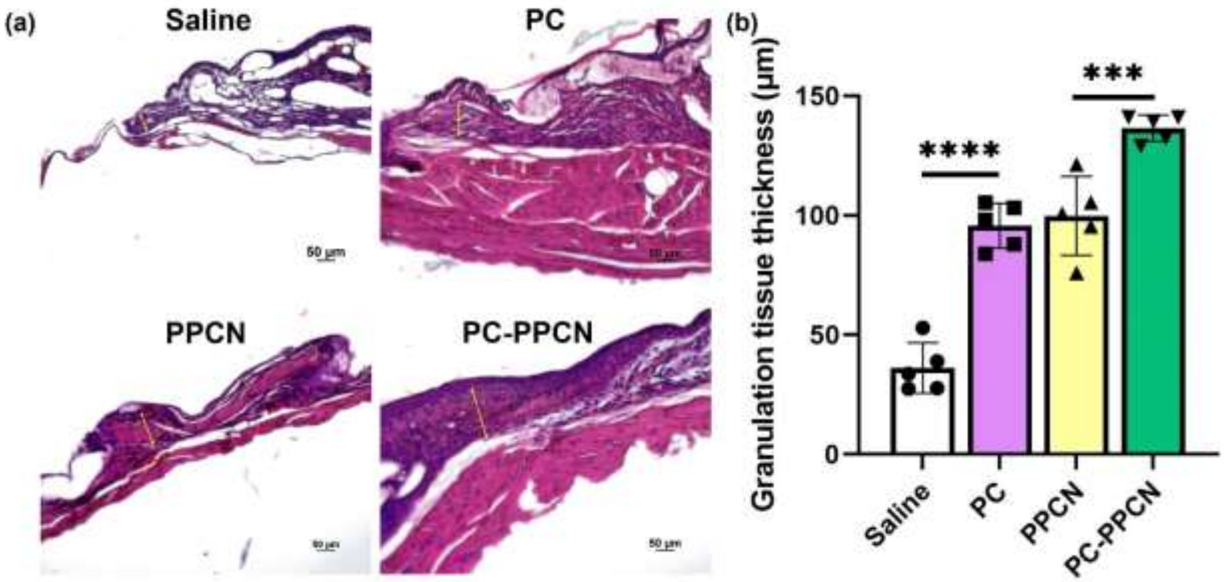

Figure S13. (a) Representative tissue sections from the wound sites collected on day 3 and stained with H&E. The yellow arrows indicate the thickness of granulation tissue. (b) Quantification of granulation tissue thickness. All data are presented as mean ± SD (n =5; ns, not significant; *P < 0.05; **P < 0.01; ***P < 0.001, ****P<0.0001).

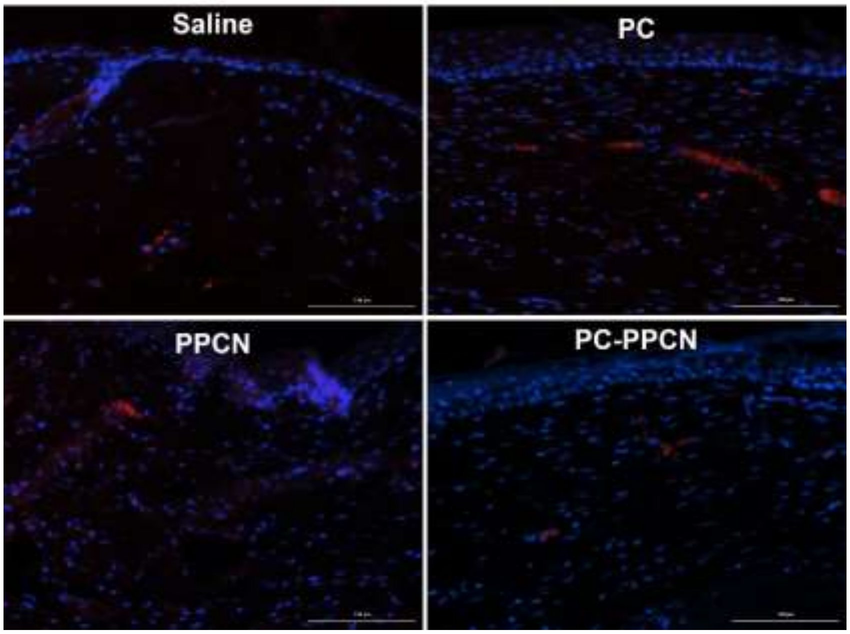

Figure S14. (a) Representative immunofluorescence images of tissues sampled at 27 days post-wounding and probed for F4/80.

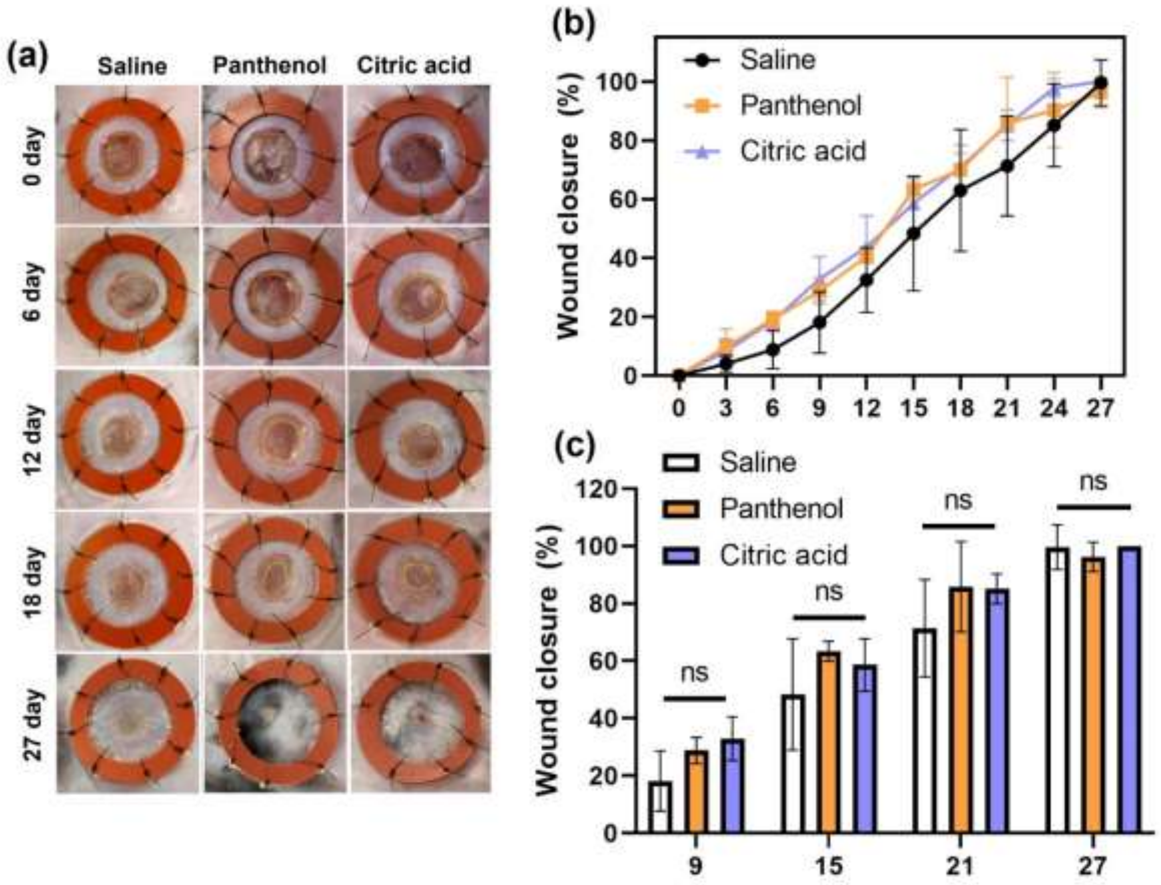

Figure S15. (a) Digital images of the wounds after treatment with saline, panthenol, and citric acid at different time points. (b) and (c) Quantification of wound areas. All data are presented as mean ± SD (n =5; ns, not significant; *P < 0.05; **P < 0.01; ***P < 0.001, ****P<0.0001).

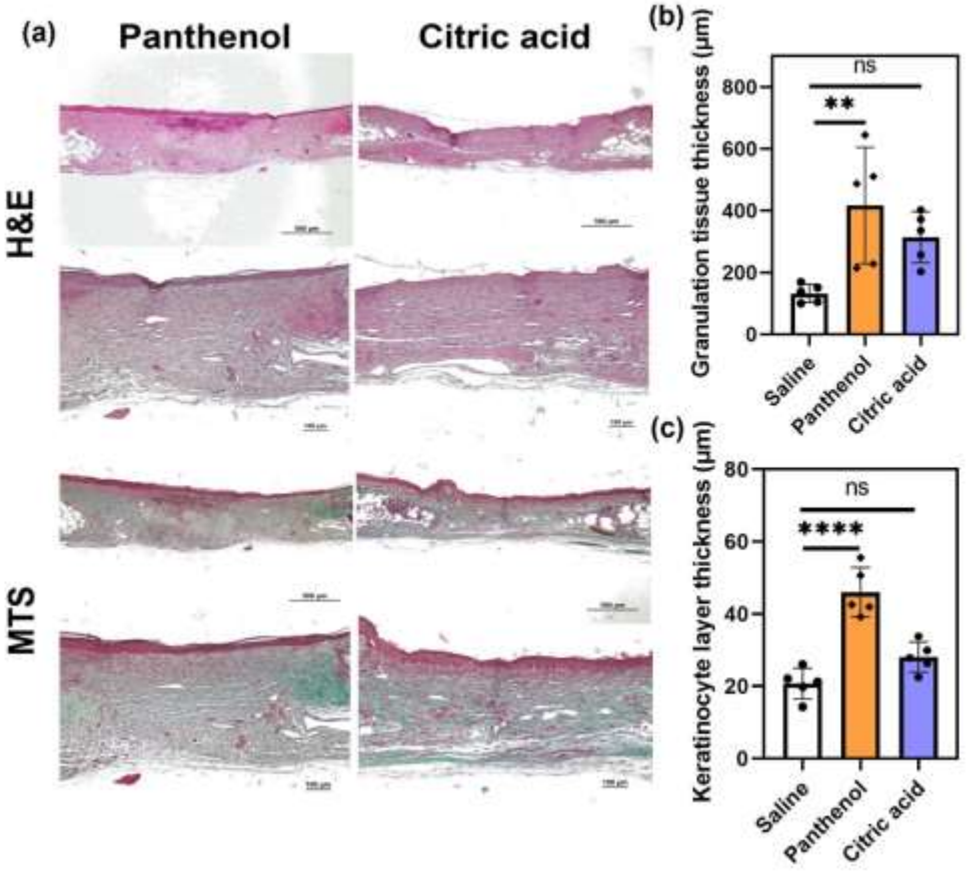

Figure S16. (a) Representative tissue sections from the wound sites were collected on day 27 and stained with H&E and MTS. (b) Quantification of granulation tissue thickness. (c) Quantification of keratinocyte layer thickness. All data are presented as mean ± SD (n =5; ns, not significant; *P < 0.05; **P < 0.01; ***P < 0.001, ****P<0.0001).

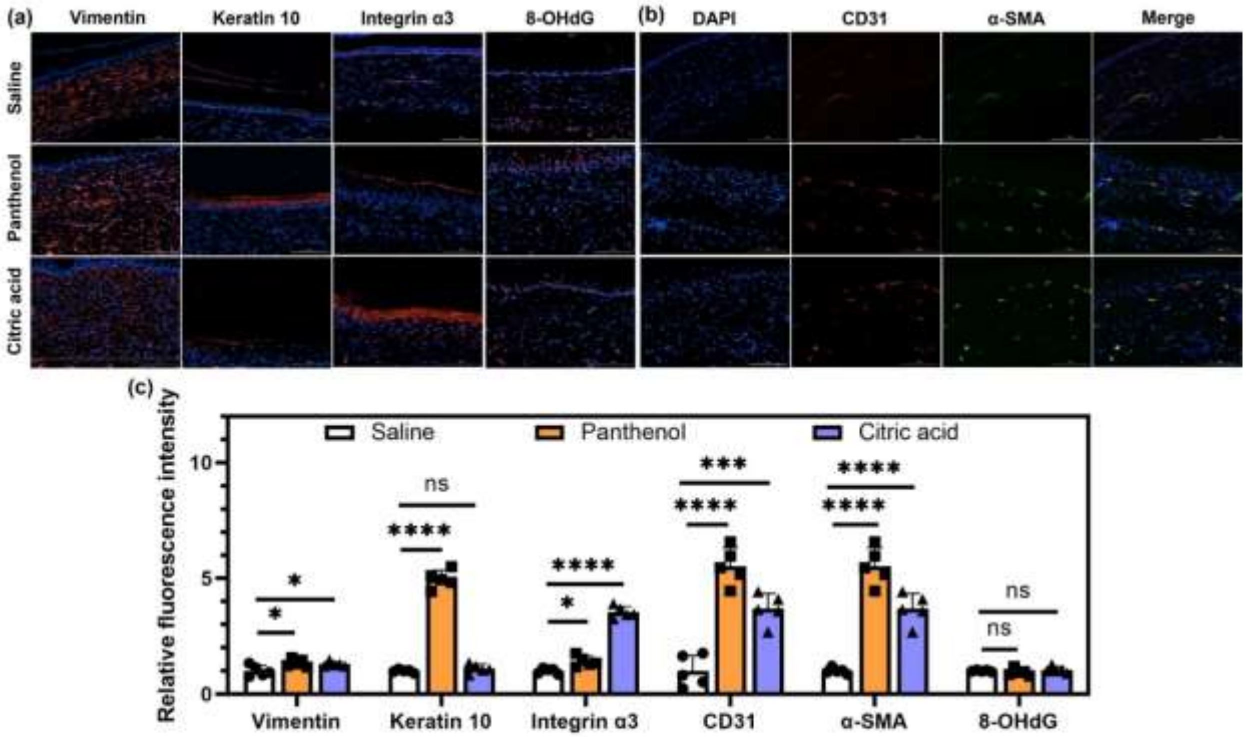

Figure S17. Representative immunofluorescence images of (a) vimentin, keratin 10, and integrin α3 and (b) CD31, α-smooth muscle actin (α-SMA), and DNA damage 8-OHdG for saline, panthenol, and citric acid 27 days post wounding. In immunofluorescence images, blue fluorescence corresponds to cell nuclei stained with 4′,6-diamidino-2-phenylindole (DAPI); green fluorescence corresponds to the expression of α-SMA, red fluorescence corresponds to the expression of vimentin, keratin 10, integrin α3, CD31, and 8-OHdG as indicated. (c) Relative fluorescence intensity from the immunofluorescence images for vimentin, keratin 10, integrin α3, CD31, α-SMA, and 8-OHdG at day 27 after treatment of saline, panthenol, and citric acid. All data are presented as mean ± SD (n =5; ns, not significant; *P < 0.05; **P < 0.01; ***P < 0.001, ****P<0.0001).

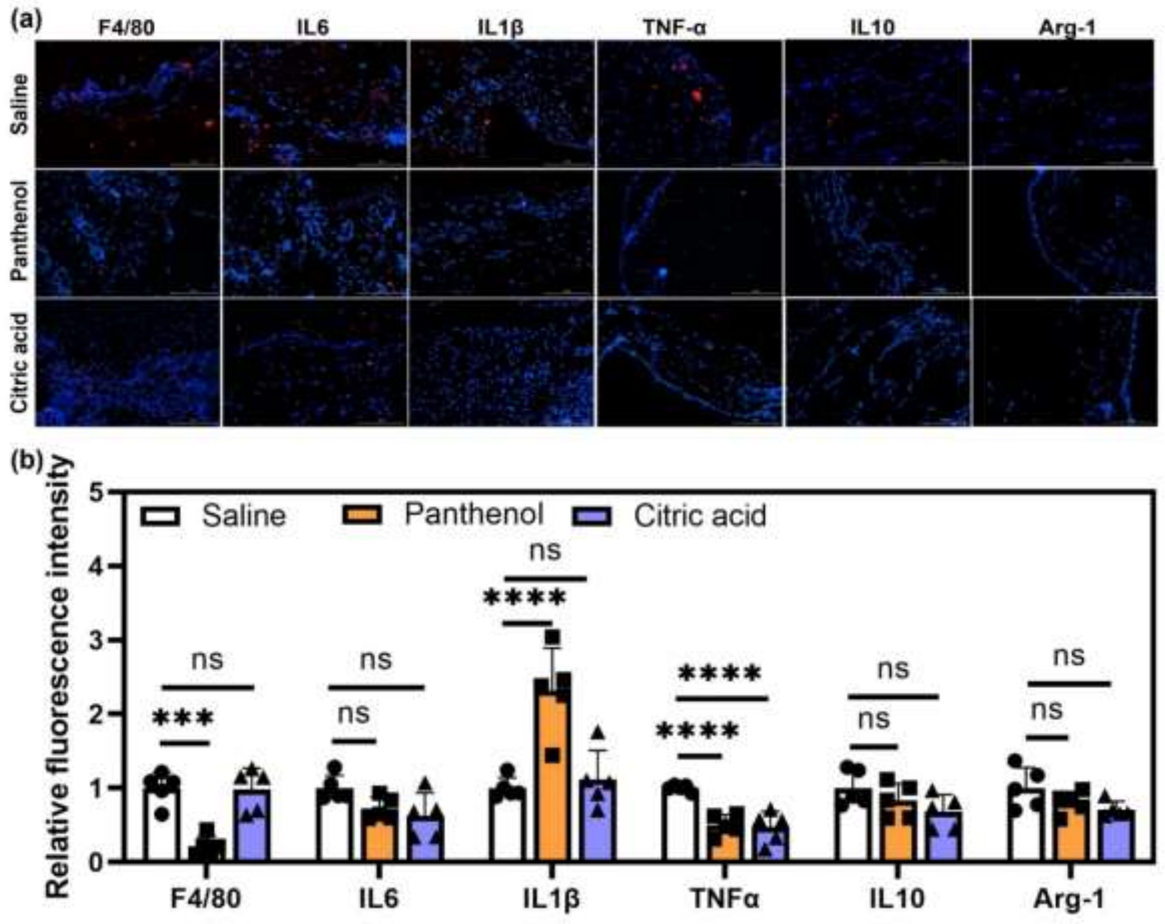

Figure S18. (a) Representative immunofluorescence images of F4/80, IL6, IL1β, TNFα, IL10, and Arg-1 for saline, panthenol, and citric acid 3 days post wounding. In immunofluorescence images, blue fluorescence corresponds to cell nuclei stained with 4′,6-diamidino-2-phenylindole (DAPI); red fluorescence corresponds to the expression of F4/80, IL6, IL1β, TNF-α, IL10, and Arg-1 as indicated (b) Relative fluorescence intensity from the immunofluorescence images for F4/80, IL6, IL1β, TNF-α, IL10, and Arg-1 at day 3 after treatment of saline, panthenol, and citric acid.

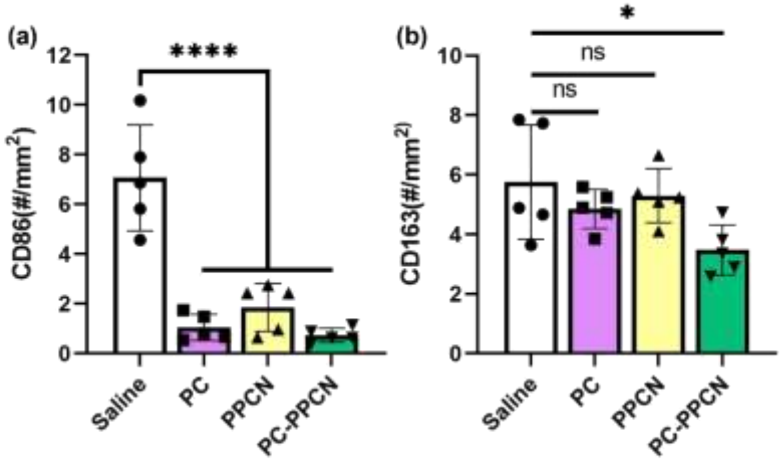

Figure S19. Quantification of CD86$^+$ and CD163$^+$ cell density. All data are presented as mean ± SD (n =5; ns, not significant; *P < 0.05; **P < 0.01; ***P < 0.001, ****P<0.0001).

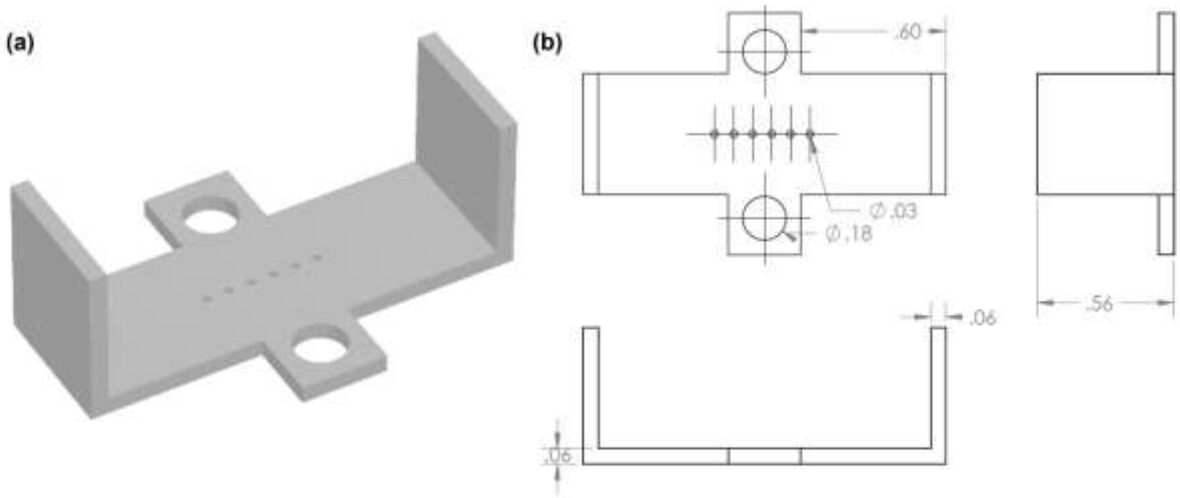

Figure S20. (a) The 3D-printed apparatus was used in the EIS measurement to secure the skin sample. (b) The dimensions of the apparatus were shown in inches.

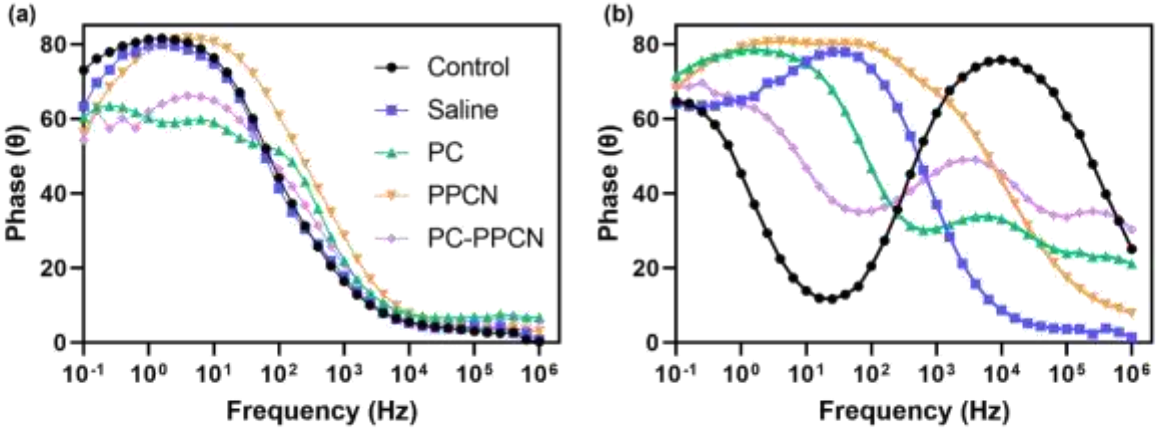

Figure S21. The phase angle as a function of frequency in EIS measurements (a) direct measurements and (b) capacitive measurements.

Supplementary Video 1. Application of PC-PPCN to the wound as a liquid that transitions to a conformal gel at body temperature.
